# Facial Kinship Verification: A Comprehensive Review and Outlook

**DOI:** 10.1007/s11263-022-01605-9

**Published:** 2022-04-19

**Authors:** Xiaoting Wu, Xiaoyi Feng, Xiaochun Cao, Xin Xu, Dewen Hu, Miguel Bordallo López, Li Liu

**Affiliations:** 1 University of Oulu, Oulu, Finland; 2 National University of Defense Technology, Changsha, China; 3 Northwestern Polytechnical University, Xi’an, China; 4 VTT Technical Research Centre of Finland, Oulu, Finland; 5 Sun Yat-sen University, Guangzhou, China

**Keywords:** Kinship verification, Facial analysis, Metric learning, Deep learning, Feature extraction

## Abstract

The goal of Facial Kinship Verification (FKV) is to automatically determine whether two individuals have a kin relationship or not from their given facial images or videos. It is an emerging and challenging problem that has attracted increasing attention due to its practical applications. Over the past decade, significant progress has been achieved in this new field. Handcrafted features and deep learning techniques have been widely studied in FKV. The goal of this paper is to conduct a comprehensive review of the problem of FKV. We cover different aspects of the research, including problem definition, challenges, applications, benchmark datasets, a taxonomy of existing methods, and state-of-the-art performance. In retrospect of what has been achieved so far, we identify gaps in current research and discuss potential future research directions.

## Introduction

Facial Kinship Verification (FKV) refers to automatically determining whether two individuals have a kin relationship or not from their given facial images or videos. Typical kinship categories include Father-Son (FS), Father-Daughter (FD), Mother-Son (MS), and Mother-Daughter (MD). As an emerging, important, and challenging problem in computer vision, FKV has attracted increasing attention, especially during the past few years. This is evidenced by the emergence of kinship verification competitions such as the Kinship Verification in the Wild (KVW) (Lu et al., 2014a, 2015), numerous workshops (RFIW2017, 2017; RFIW2018, 2018) and tutorials (Robinson et al., 2019) focusing on the topic, and the increasing number of methods proposed (Dahan and Keller, 2020; Lu et al., 2014a; Wang et al., 2020; Yan et al., 2014a; Zhang et al., 2015).

**Fig. 1:**
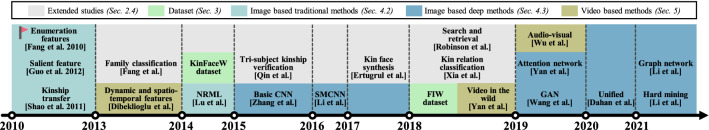
The milestones of FKV methods. The figure shows the evolution of facial kinship verification study. The first facial kinship verification study was carried out in 2010. After 2010, much attention was attracted to FKV research. We sort these studies from aspects of (1) Image-based FKV using traditional methods, which are reviewed in Sect. 4.2; (2) FKV from images using deep methods (Sect. 4.3); (3) Video-based kinship verification methods (Sect. 5); (4) Extended studies (that are reviewed in Sect. 2.4); and (5) Important kinship datasets (reviewed in Sect. 3). Before 2015, the main studies are based on traditional methods (Fang et al., 2010b; Guo and Wang, 2012; Lu et al., 2014c; Shao et al., 2011a). In 2015, the deep learning method (Zhang et al., 2015) was proposed. Video-based FKV study dates back to 2013 (Dibeklioglu et al., 2013), while it has drawn very little attention until 2018 that (Yan and Hu, 2018b) proposed FKV from unconstrained videos. From 2013 to 2019, multiple extended kinship topics emerged  (Ertugrul and Dibeklioglu, 2017; Fang et al., 2013b; Qin et al., 2015a; Robinson et al., 2018; Xia et al., 2018)

There are at least four reasons that explain this trend. The first is due to its various potential applications. In the anthropology and genetics domain, FKV can help to study the hereditary characteristics of close relatives in social relationships (M’charek, 2020). In the field of public social security, it can be applied to finding missing children, border control and customs, and criminal investigations (Kohli et al., 2019b; Lu et al., 2014c). In the social media domain, the FKV can be used for family photo album organization, improving the performance of face recognition systems and social media analysis (Lu et al., 2014a). In addition, FKV also has potential applications in smart homes, the Internet of Things (IoT) (Jang et al., 2017) and personalization. The second reason is that the FKV serves as a fundamental study among visual kinship problems, such as family recognition, family retrieval (Robinson et al., 2018). The third point is the low sensory perception of human eyes to quantify the similarity of two images from different people (Bordallo López et al., 2018). Features such as the distance between the eyes and the shape, color, and size of the facial parts are not easily judged at a glance, resulting in low recognition accuracy. Finally, the FKV problem attracts researchers from diverse disciplines such as computer vision, machine learning, pattern recognition (Li et al., 2021a), anthropology, psychology, and neuro science (Clemens and Brecht, 2021; Kohli et al., 2018), and provides a cross-fertilization ground for stimulating cross-discipline studies.

Before the FKV research started in the field of computer vision, kinship has been widely studied in the field of psychology (Alvergne et al., 2014; Dal Martello and Maloney, 2006, 2010; DeBruine et al., 2009; Maloney and Dal Martello, 2006). Researchers concluded that humans could infer kinship through visual clues, in particular, based on facial resemblance. The research on automatic FKV dates back to the work in 2010 (Fang et al., 2010b) by Fang *et al*.. At the beginning of kinship research, shallow features such as facial geometry or the color of the eyes were used for determining the kinship (Fang et al., 2010b). In 2014, Lu et al. (2014a) introduced the metric learning scheme for solving the kinship verification problem. Following that, kinship research received a wide range of attention. In 2015, Zhang et al. (2015) applied deep learning approaches in kinship verification, which also brought the FKV into the deep learning era (Dahan and Keller, 2020; Li et al., 2016, 2020; Wang et al., 2020; Yan and Wang, 2019). Meanwhile, the emergence of the large-scale kinship dataset FIW (Robinson et al., 2018) promoted the further development of the field. Based on different application scenarios, FKV research has been extended to multiple complementary topics (Ertugrul and Dibeklioglu, 2017; Fang et al., 2013b; Qin et al., 2015a; Shao et al., 2011a; Wu et al., 2019; Xia et al., 2018). The milestones above are listed in the Fig. 1 by year.

Image-based kinship verification has advantages such as ease of computation and available normalized and constrained data, such as those contained in datasets depicting identity pictures. On the other hand, it lacks temporal information. In recent years, research has been extended to include also video-based facial kinship verification. The recent video-based kinship verification can integrate information based on the facial dynamics (Dibeklioglu et al., 2013; Dibeklioğlu et al., 2012a), although the motion blur and the low face resolution in some video material increase the problem’s difficulty. Additional modalities such as voice information, usually accompanying videos, can reinforce the information of facial features (Wu et al., 2019).

Given this period of rapid development, to stimulate future research, the goal of this survey is to provide a comprehensive overview of FKV from image-based and video-based aspects. On this young topic studied for about one decade, only a limited number of existing surveys can be found. Dandekar and Nimbarte (2014) conducted the first survey on FKV and mainly reviewed earlier methods prior to 2014. The later review given by Wu et al. (2016b) in 2016 covers mainly traditional methods before deep learning and is fairly short. The review by Almuashi et al. (2017) in 2017 focused on the derivation, definition, significance, and challenges of the FKV problem, but barely discussed various methods. Georgopoulos et al. (2018) reviewed kinship verification from the aspect of face aging in 2018, mainly pointing out the inherent synergy between kinship and aging, and challenges derived by this phenomenon, rather than offering a systematic review of FKV. Although the review by Qin et al. (2020) is recent, it categorizes kinship recognition methods into feature extraction and metric learning methods and covers few about recent deep learning-based approaches. Very recently, the excellent survey by Robinson et al. (2021) focused on many kinship recognition tasks (including the problem of FKV) and practical issues, while missing the details, connections, and performance comparison of various FKV methods. Among various visual kinship recognition tasks, FKV is the most popular one and significant success has been achieved. At this stage, there is a need for a thorough review of FKV, promoting further development, particularly for researchers wishing to enter the field.

**Fig. 2:**
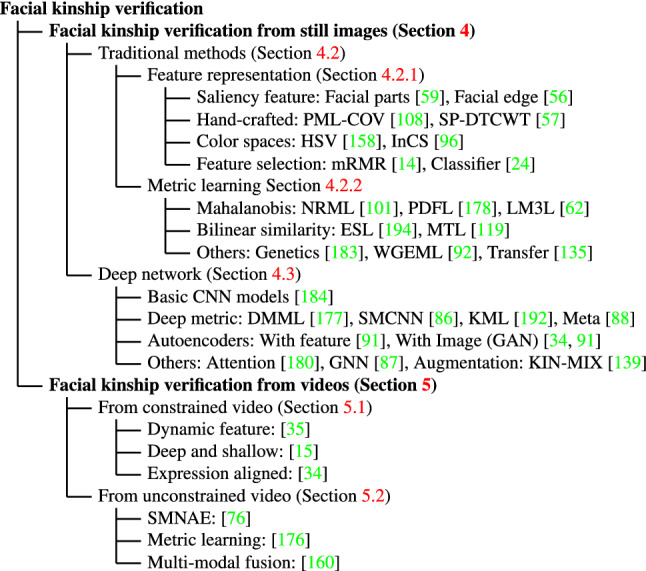
A taxonomy of facial kinship verification methods

Since the existing surveys on FKV are still not comprehensive enough, the goal of this survey is to provide a comprehensive overview of FKV. The main contributions in this survey are summarized as follows:

- We provide a comprehensive overview on facial kinship verification methods from both facial images and facial videos. This survey also includes a summary of challenges, current developments, including datasets, representative methods and SOTA performance.
- We build an intuitive taxonomy and situate past research works in relation to each other.
- New ideas and insightful thoughts derived from the current review are provided for developing the next generation of kinship verification techniques.

**Fig. 3:**
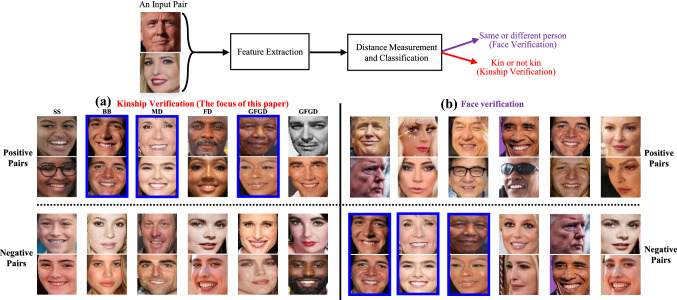
General pipeline for face verification task and kinship verification task. Both tasks calculate the similarity of two facial images. While positive pairs in kinship verification task are negative pairs in the case of face verification

The rest of this survey article is organized as follows. The problem definition, challenges, kinship verification from human perception, and kin-related topics are summarized in Sect. 2. In Sect. 3, we review the kinship datasets and compare their attributes from multiple perspectives. Starting from Sect. 4 to Sect. 5, we review and discuss the published kinship verification methods. A taxonomy of these methods is illustrated in Fig. 2. Section 4 summarizes the image-based kinship verification, including traditional methods Sect. 4.2 and recent deep learning methods (Sect. 4.3). In Sect. 5, we review video-based kinship verification. Section 6 summarizes and compares the typical method performance and analyzes the key influence factors. In Sect. 7, we conclude the paper and discuss the possible promising future research directions.

## Background

### The Problem

Given a pair of facial images, the objective of kinship verification is to judge whether two people are biologically related (with a typical kin relation). Specifically, the current kinship verification research uses a clear distinction of multiple kin relation types to study the verification problem. Only close family relationships are involved. These kin relations can be categorized into three levels of generation, *e.g., *Siblings, Parent-Child, and Grandparent-Grandchild.^1^ The four parent-child relations attract the most attention (Lu et al., 2014a), mainly because of their application value. Kinship verification can be formulated as a binary classification problem (Kin vs. Non-kin). FKV primarily consists of two critical sub-problems: feature extraction and classifier designation. Formally, as shown in Fig. 3, given a pair of faces $(\mathbf{X} ,\mathbf{Y} )$^2^, appropriate feature representations $(\phi (\mathbf{X} ),\phi (\mathbf{Y} ))$ are extracted from both images, and then a classifier is used to determine if the two faces have a kin relationship or not.

In order to better understand the FKV problem, we would like to point out the relationship between two similar problems: the FKV problem and the face verification problem (face pair matching) (Zhao et al., 2003) which are contrasted in Fig. 3. As can be seen from Fig. 3, both problems share a similar algorithm pipeline. The classification at the end is used to judge if two faces are the same individual or not in the case of face verification, or if they have a kin relation or not in the case of kinship verification. Intuitively, both problems depend on the existence of similar facial cues for making judgments (Hansen et al., 2020; Krupp et al., 2008), especially in the case of face verification where each positive pair represents the same individual (see Fig. 3b). In the case of kinship verification, each positive pair represents two different individuals with a kin relation (detecting kin clues in specific areas of the face rather than from the entire face Dal Martello and Maloney, 2006). Note that all positive pairs (including identical twins) in the case of kinship verification (see Fig. 3a) are negative pairs for face verification. It is interesting to ask a question: Do pairs of the same individuals (positive pairs in the case of face verification) belong to positives or negatives in the case of kinship verification?^3^ This question has been overlooked as current FKV research assumes each input face pair belongs to two different individuals in their experimental setting. From an anthropological point of view, FKV is based on the degree of genetic similarity between the faces of two subjects. Thus, it is reasonable to expect that an FKV system will give high prediction accuracy for facial pairs of the same person. When facial images from one individual present age variation, the study of age-invariant face verification can be somehow viewed as self-kinship verification, where the system verifies the same individual as related to himself  (Kohli, 2019; Lu et al., 2014a). On the other hand, as facial aging and kinship are both genetically inherited (Georgopoulos et al., 2018), kinship is capable of providing guidance for age progression and boost face verification. Conversely, a de-aging process can be performed to learn discriminative identity features for both face verification (Xu et al., 2017) and kinship verification (Wang et al., 2018).

Kinship is a well-established biological concept, but determining what kind of similar facial cues are critical for FKV is still an open question. According to recent psychology studies (DeBruine et al., 2009; Hansen et al., 2020), facial similarity and kinship judgments are highly correlated but not strictly synonymous. This makes FKV a difficult problem with various challenges which we discuss below.

**Fig. 4:**
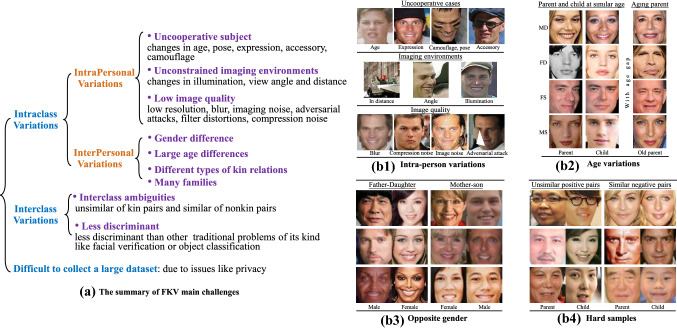
The main challenges of facial kinship verification. Sub-figure **a** provides a taxonomy of these challenges brought by intraclass variations, interclass variations, and data establishment. The right sub-figure illustrates key scenarios with facial sample images. In the right, **b1**, **b2** as well as **b3** show intraclass variations, in which **b1** contains the possible variations within one subject, with each image line demonstrating influences from different factors. Then, **b2** and **b3** illustrate the facial similarity gap between kinship caused by age and gender differences, as well as variations among kin pairs and families. Figure **b4** demonstrates less discrimination of FKV that hard kin and non-kin samples exist when kin pairs have less similarity on appearance, while non-kin pairs inversely show significant similarity

### Main Challenges

As we defined above, FKV is formulated as a binary classification problem. The difficulty of FKV stems partially from the fact that the kinship facial pairs do not belong to the same identity and only show hidden genetic facial similarities that are more complex and less discriminative than similarities in other problems like facial verification. As discussed above, it is evident from psychology research (DeBruine et al., 2009; Hansen et al., 2020) that facial similarity and kinship judgments are not strictly synonymous though highly correlated, which makes the problem of FKV even harder. The main challenges of FKV are summarized in Fig. 4, with visual examples for illustration.

(1) *Large intraclass variations* As can be seen from Fig. 4a, there are two types of intraclass variations: *intrapersonal* variations (facial appearance changes of the same identity) and *interpersonal* variations (facial appearance differences of different identity). The large intrapersonal variations come from uncooperative subjects such as changes in age pose, expression and accessories, unconstrained imaging environments like changes in illumination, imaging distance and angle, variations in image quality and resolution, blur, and even adversarial attacks (Fig. 4b1). All these pose great challenges for extracting discriminative features for kinship verification and greatly impact FKV performance. Many early approaches for FKV only considered facial images acquired in cooperative conditions. Therefore, it is more practical to build large-scale kinship datasets in the wild.

As the input of an FKV algorithm is a pair of facial images belonging to two individuals, the goal of FKV is to explore the hidden factors of visual similarity between the two input faces for kinship determination. Therefore, there are significant interpersonal variations that increase the intraclass distance between the positive class samples. Firstly, there can be a significant age gap between the kin pairs, particularly when verifying cross-generation kinship types. Figure 4 (b2) shows parent-child pairs with a similar age and a considerable age gap. It has been demonstrated that parent-child pairs with a similar age have more similarities (Hansen et al., 2020; Xia et al., 2011). However, pairs of older parents and younger children can have significant textural differences between the two faces, which negatively influences similarity. Secondly, gender differences also negatively influence facial similarity. As shown in Fig. 4b3, kin pairs of mother-son, father-daughter, and brother-sister have different gender variations. It has been shown that non-kin pairs with same gender have more similarities than those with different gender (Hansen et al., 2020). Finally, in addition to the existing considered kinship types, facial similarities can also exist between some family members when one increases the height or width of the family tree (e.g. by including cousins and nieces). One reason for this is that the inheritance among different kinship types is not deterministic (Monks et al., 2004). It is tough to determine a mathematical inheritance model due to its randomness and requirement on multidisciplinary knowledge (Alvergne et al., 2007; Monks et al., 2004).

(2) *Small interclass variations* As we defined above, the FKV aims to learn a binary classifier by distinguishing a number of positive kinship pairs from a number of negative samples. The similarity among kin faces attributes to hidden factors instead of the whole face. As illustrated in Fig. 4b4, some positive examples may have small similarities, whereas negative examples may have high similarities. Therefore, small positive and negative variations decrease the interclass separation and pose significant challenges for learning the real decision boundary. In addition, there is a severe imbalance issue (Li et al., 2021b), *i.e., *, the number of negatives is significantly more than the number of positive pairs.

(3) * Difficulty in gathering large-scale kinship datasets* The lack of large kinship datasets impedes the development of FKV algorithms, especially the development of deep learning-based methods which are data-hungry. It is essential to collect a large kinship dataset that can represent the actual data distributions of families worldwide, reflecting the intraclass and interclass variations discussed above. However, due to security and privacy issues, it is challenging to meet this requirement.

### Facial Kinship Verification Based on Human Perception

Human has an instinctive perception ability to indicate the familial genetic relatedness between individuals (Maloney and Dal Martello, 2006). The earliest research on demonstrating human’s ability to recognize kinship from faces dates back to 1991 (Porter, 1991). To provide a clear and high-level understanding of FKV, in this subsection, we focus on reviewing research results on human visual perception of kinship.

#### Psychological Issues Relevant to Facial Kinship Verification

Psychology research on FKV studies kinship verification procedure from human perspective. This knowledge has a special significance since it can guide automatic FKV research and support the understanding of the experimental results. Recent psychological studies examine multiple factors, such as facial variances (Dal Martello et al., 2015; Dal Martello and Maloney, 2010; Fasolt et al., 2018), subject’s gender, age (DeBruine et al., 2009; Hansen et al., 2020) and kin regions (Alvergne et al., 2014; Dal Martello and Maloney, 2006), that how those factors affect the human ability to verify kinship.

The effects of facial variance in kinship verification, including rigid deformation (*e.g., *facial image rotation) and non-rigid deformation (*e.g., *facial expressions) have been studied. Dal Martello et al. (2015) found that kinship verification by humans is not significantly influenced by face inversion since the judgment mainly relies on geometry similarity other than invertible attribute cues. When faces show non-rigid deformations (e.g., different facial expressions), they can negatively impact kinship verification and reduce the accuracy of verification (Fasolt et al., 2018), compared with neutral face images.

More specifically, Dal Martello and Maloney (2010) conducted experiments by showing partially occluded facial images to participants to explore the kin cue distribution in facial regions. They concluded that the left and right parts of the face contributed equally to kinship verification and only showed slightly less kin information than the entire face. Dal Martello and Maloney (2006) in 2006 and (Alvergne et al., 2014) in 2014 found that the upper half of the face contains a large amount of kinship information compared with the lower face, as the mouth area is prone to noise effects due to its morphological variation. Alvergne et al. (2014) also notes that kinship cues depend on specifically effective facial areas rather than the entire facial area.

Regarding the individual’s biological attributes, researchers(DeBruine et al., 2009; Hansen et al., 2020) pointed out that gender and age differences can significantly reduce the accuracy of kinship verification due to the feature difference brought by gender and age.

#### Human Performance on Public Kinship Datasets

Table 1 summarizes the human performance on the public kinship datasets. In the experiments, volunteers were given a pair of kin images/videos and asked whether they have a kin relation. As it can be seen from the table, kin relations with different gender (FD, MS) show lower verification accuracy when compared with kin relations of the same gender (FS, MD). As for age variance, on UVA-NEMO Smile dataset, kin pairs from the same generation (BB, SS, BS) share more kin resemblance for the human perception (Alvergne et al., 2014; Dal Martello and Maloney, 2006). The effect of age and gender show consistency with earlier psychological research results. This phenomenon has also been demonstrated by Bordallo López et al. (2018) that humans showed a tendency to assess better brothers and sisters, especially when they are of the same gender, than parents and children.

As a particular note, on the TSKinFace dataset, when volunteers are provided images of both parents, the participants can give more accurate judgments. In most of the experiments, the participants could take advantage of prior knowledge (*e.g., *ethnicities, age differences, and well-known celebrities Bordallo López et al., 2018) rather than relying solely on facial cues and kin similarity between faces. Recent experiments by Robinson et al. (2018) considered these biases (participants were asked to skip when they had prior knowledge of subjects) and showed a noticeable decrease in performance, which sank to $57.5\%$ on average.

On the basis of human cognitive studies, an automatic kinship verification system was developed as demanded as the human brain has limitations on making precise judgments. Computers capture the facial perceived similarity between faces objectively and quantitatively. Experimental results indecate that computer vision and machine learning methods have superior performance in kinship verification compared with human’s ability (Dibeklioğlu et al., 2012a; Lu et al., 2014a; Qin et al., 2015a; Robinson et al., 2018; Yan and Hu, 2018b).

**Table 1:** Human performance ($\%$) of verifying kinship evaluated on public datasets

Dataset	FS	FD	MS	MD
KinFaceW-I (Bordallo López et al., 2018; Lu et al., 2014c)	78.2	75.8	74.6	85.8
KinFaceW-II (Bordallo López et al., 2018; Lu et al., 2014c)	86.0	76.8	84.4	86.6
TSKinFace Qin et al., 2015a	77.3	73.5	74.2	75.5
	FM-S		FM-D	
	79.9		79.2	
FIW (Robinson et al., 2018)	57.5			
UVA-NEMO Smile (Dibeklioglu et al., 2013; Dibeklioğlu et al., 2012a)	73.3	66.7	71.7	81.5
	BB	SS	BS	
	96.2	88.7	82.8	
KFVW (Yan and Hu, 2018b)	75.0	70.5	73.0	73.5

### The Extended Studies

**Fig. 5:**
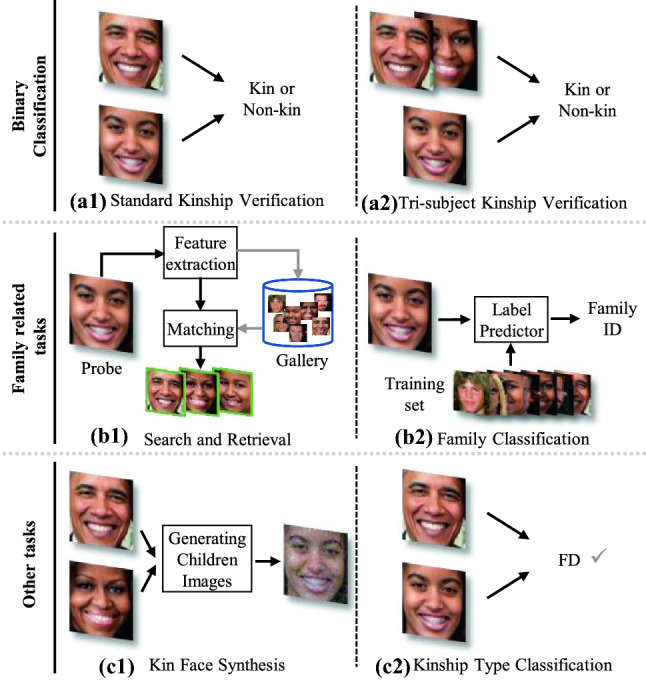
Kinship analysis tasks. The main task is kinship verification. We categorize kinship-related research directions into binary classification tasks, family-related tasks, and other tasks

FKV study is the widely explored and fundamental research problem of kinship recognition. Due to variant applications of kin-tasks, complementary kin research problems have emerged, which are illustrated in Fig. 5.

#### Tri-Subject Kinship Verification

A child’s genetic inheritance comes from both parents (father and mother). This leads to the Tri-subject kinship verification (Qin et al., 2015a), where the inputs are both parents’ facial images and the child’s facial image. Suppose that $\mathbf {X}_1$ and $\mathbf {X}_2$ represent father and mother’s facial images (or videos) respectively and $\mathbf {Y}$ formulates a child’s facial image (or video). The feature representations of parents and child are extracted, $\phi (\mathbf {X}_1)$, $\phi (\mathbf {X}_2)$ and $\phi (\mathbf {Y})$. The distance are computed between a child and his or her parents, $d(\left\langle \phi (\mathbf {X}_1),\phi (\mathbf {X}_2) \right\rangle ,\phi (\mathbf {Y}))$, to verify whether they have a kin relation. Tri-subject kinship verification is also a binary classification problem.

#### Family Classification

Family classification (Fang et al., 2013b) is a multiclass classification problem, *i.e., *the classification task contains multiple categories, and each category represents a family. Given a pending facial image, we need to determine which family it belongs to. A collection of *k* families is represented by $\chi =\left\{ \mathbf {X}_{1}, \mathbf {X}_{2}, \ldots , \mathbf {X}_{k}\right\}$. The corresponding multiclass label can be written as $\left\{ y_{1}, y_{2}, \ldots , y_{k}\right\}$. By training a classifier, the system outputs the family label of an input facial image $\mathbf {x}$. The difficulty of family classification increases when family classes increase. In the FIW dataset (Robinson et al., 2018), family classification accuracy is only 16.18% from a total of 564 families.

#### Family Search and Retrieval

Family search and retrieval (Robinson et al., 2018) is designed to match family members to the input facial image, where the search is performed on a set consisting of members from all families. The input facial image is a query, and the output gives the most matched *K* family members. The difference between family classification and family retrieval is that family classification focuses on the training of family classification models, and family retrieval tries to retrieve face images that are more similar to the images to be queried through similarity metric learning and find the input’s parents and other kinship members.

#### Other Tasks

Other tasks include kin face synthesis (Ertugrul and Dibeklioglu, 2017; Gao et al., 2019) and kin relation classification (Wang et al., 2020; Xia et al., 2018). Kin face synthesis study takes the facial images of parent(s) to synthesize the child’s image. By synthesizing the kid’s facial image, kinship data are augmented for training and improve the model consistency, thus assisting the FKV. Besides, it can also be applied in the matching of missing children. In kin relation classification, the inputs are two facial images with a particular kin relation and the system estimates which specific kin relation they have. This task has applications in family album organization and social media analysis.

Since the study of kinship analysis is still during its initial stages, facial kinship verification is the key and core of kinship research, which is also the focus of this survey.

## Datasets and Evaluation Metrics

It has been widely accepted that quality datasets, especially in the big data era, play an essential role in the research. The problem of FKV is no exception. Benchmarking datasets not only serve as a common ground for performance measurement and comparison of various algorithms, but also help the field to progress towards increasingly complex and challenging problems. Therefore, in this section, we first review the existing public datasets for FKV with motivations, statistics, available sites, and the supported kinship recognition problems, then discuss evaluation metrics, and finally summarize the findings.

### Datasets

Currently, there are twelve commonly used kinship datasets. The attributes of those datasets are summarized in Table 2. We will introduce them in the following from image and video categories.

**Table 2:** The summary of characteristics for kinship datasets. (Some abbreviations

	Dataset	Year	Size	Resolution	M	A	F	C	Contribution	Targeted study
Image	CornellKin (Fang et al., 2010b)	2010	150 pairs	100$\times$100	$\times$	$\times$	$\times$	$\times$	The first kinship dataset	1V1
	UB Kinface (Shao et al., 2011a; Xia et al., 2011)	2011	200 groups	89$\times$96	$\times$	$\checkmark$	$\times$	$\times$	Images of young,old parents	1V1, kinship transfer
	Family 101 (Fang et al., 2013b)	2013	101 family trees	120$\times$150	$\checkmark$	$\checkmark$	$\checkmark$	$\times$	With family structure	1V1, family tasks
	KinFaceW-I (Lu et al., 2014c)	2014	533 pairs	64$\times$64	$\times$	$\times$	$\times$	$\times$	From different photos	1V1
	KinFaceW-II (Lu et al., 2014c)	2014	1000 pairs	64$\times$64	$\times$	$\times$	$\times$	$\times$	From same photos	1V1
	TSKinFace (Qin et al., 2015a)	2015	1015 groups	64$\times$64	$\times$	$\times$	$\checkmark$	$\times$	Both parents’ facial images	2V1
	FIW (Robinson et al., 2018)	2016	1000 families	224$\times$224	$\checkmark$	$\checkmark$	$\checkmark$	$\times$	The largest kinship dataset	1V1, 2V1, family tasks
	WVU (Kohli et al., 2016)	2017	113 pairs	32$\times$32	$\checkmark$	–	$\times$	$\times$	Each one has four images	1V1
Video	UvA-NEMO Smile (Dibeklioğlu et al., 2012a; Dibeklioglu et al., 2013)	2012	1240 videos	1920$\times$1080	$\checkmark$	$\times$	$\times$	$\checkmark$	First video kinship dataset	1V1
	KFVW (Yan and Hu, 2018b)	2018	418 pairs	900$\times$500	$\times$	$\times$	$\times$	$\times$	First uncontrolled video dataset	1V1
	FFVW (Sun et al., 2018)	2018	100 groups	-	$\times$	$\times$	$\checkmark$	$\times$	Video tri-subject	2V1
	KIVI (Kohli et al., 2019b)	2019	211 families	-	$\times$	$\times$	$\checkmark$	$\times$	Uncontrolled video dataset	1V1
	TALKIN (Wu et al., 2019)	2019	400 pairs	1920$\times$1080	$\times$	$\times$	$\times$	$\times$	Multi-modal kinship dataset	1V1

Column ’M’, ’A’, ’F’, ’C’: Multiple samples (M), Age variety (A), Family structure (F), Controlled environment (C). Column ’Targeted study’: Facial kinship verification (1V1), Tri-subject kinship (2V1), applicable also to Table 3)

#### Image Datasets

*Cornell KinFace* dataset (Fang et al., 2010a, b) is the first public kinship dataset collected from the Internet. It had pioneering significance for the development of kinship datasets in providing a solid reference for the establishment of other larger and more complex kinship datasets.

*UB KinFace* dataset (Shao et al., 2011a, b; Xia et al., 2011) is the first kinship dataset that includes children’s, young parents’ and old parents’ facial images. The hypothesis is that children would have a more similar appearance with their young parents. Thus young parent image can serve as a bridge between old parent and children.

*KinFaceW* dataset (Lu et al., 2014b, c) has two subsets, namely as KinFaceW-I and KinFaceW-II. Both of them are collected from the Internet. The difference between them is that the kin images in KinFaceW-I are cropped from different photos, and the kin images of KinFaceW-II are from the same photo. KinFaceW dataset has been widely used in kinship verification research.

*Family 101* (Fang et al., 2013a, b) is the first kinship dataset that has a family tree structure. Most of the facial images are in grayscale.

*TSKinFace* dataset (Qin et al., 2015a, b) is mainly used for the study of tri-subject kinship verification. TSKinFace has two types of kinship relations: Father-Mother-Son and Father-Mother-Daughter. The facial images are all downloaded from the Internet.

*WVU* (Kohli et al., 2016, 2017) dataset has variations on each individual, and each person has four facial images.

*FIW* (Families In the Wild) (Robinson et al., 2016, 2018) is the largest and most comprehensive kinship dataset by far. FIW dataset is organized by the family tree structure. It consists of multiple facial images from different periods for each family member. Regarding kin relations, FIW has relations of same generation, first generation, and second generation. FIW is similar to Family 101, but it is much superior in aspects of family structure, data volume, and data variants.

#### Video Datasets

*UVA-NEMO Smile* dataset  (Dibeklioglu et al., 2013; Dibeklioğlu et al., 2012a, b) was first established aiming at classifying spontaneous smiles and deliberate smiles. Because the participants in the dataset are family-related, it is also considered as the first video-based kinship dataset. All facial videos are smiling videos collected under indoor constrained conditions.

*KFVW* (Kinship Face Videos in the Wild) dataset (Yan and Hu, 2018a, b) was proposed in 2018. The difference from the UVA-NEMO Smile dataset is that KFVW is collected under natural varying environments. Videos have no constrains on illumination, pose, occlusion, background, expression, or age. These videos are collected from the Internet.

*FFVW* dataset (Sun et al., 2018) is similar to the image kinship dataset TSKinFace, which is mainly used to study the problem of tri-subject kinship verification. All these facial videos are from the Internet and collected under an unconstrained natural environment.

*KIVI* dataset (Kohli et al., 2019a, b) is organized with the family structure containing facial videos of 503 subjects from 211 families. The dataset is downloaded from the Internet.

*TALKIN* dataset (Wu et al., 2019) is the very first multimodal kinship dataset, which consists of both facial video and audio modalities for each subject. All the videos are facial videos with the subject talking. The videos are downloaded from YouTube.^4^

#### Evaluation Metrics

In kinship verification experiments, the data is usually divided into positive pairs and negative pairs. The positive pairs are all pairs with kin relations in the dataset, while negative pairs are most often generated randomly among the image pairs without a kin relation. Generally, when establishing a protocol, the number of positive pairs and negative pairs is created balanced, although the creation of additional negative pairs has also been explored (Li et al., 2021b). The most typical evaluation protocols are based on N-fold cross-validation with the intent to reduce overfitting. In the most typical 5-fold configuration, four folds are used as training data, while the remaining one is used for testing. After repeating the process through all five testing folds, we can compute the final result with the average accuracy of each one of the five. Notably, in this configuration, the positive and negative pairs should only be generated within each fold.

Verification accuracy is the typical assessment criteria in kinship verification studies. Given True Positive (TP), True Negative (TN), False Positive (FP), and False Negative (FN). The accuracy *A* is obtained by:

1 $$\begin{aligned} A=\frac{TP+TN}{P+N} \end{aligned}$$

### Kinship Dataset-Based Worldwide Competitions

Several kinship competitions were held based on the public datasets, KinFaceW and FIW. Two series of FKV competitions were held, KVW and RFIW. Two KVW competitions were held in years of 2014 and 2015 on the KinFaceW dataset. The task is kinship verification from facial images. The RFIW competitions were held in recent years on FIW with different sub-tasks. Table 3 summarized these competitions.

**Table 3:** The summary of kinship competitions

Year	Competition	Dataset	Tasks				Platform
			1V1	2V1	FC	SR	
2014	KVW (Lu et al., 2014a)	KinFaceW	$\checkmark$				IJCB
2015	KVW (Lu et al., 2015)		$\checkmark$				FG
2017	RFIW (Robinson et al., 2018; RFIW2017, 2017)	FIW	$\checkmark$		$\checkmark$		ACM MM
2018	RFIW (RFIW2018, 2018)		$\checkmark$		$\checkmark$		FG
2019	RFIW (RFIW2019, 2019)		$\checkmark$		$\checkmark$		FG
2019	RFIW (RFIW2019-Kaggle, 2019)		$\checkmark$				Kaggle
2020	RFIW (Robinson et al., 2020; RFIW2020, 2020)		$\checkmark$	$\checkmark$		$\checkmark$	FG

Some abbreviations. Column ’Tasks’: Family classification (FC), Search and Retrieval (SR)

### Summary and Discussion

Compared with the first dataset, the recent ones have been improved in size, structure, kin relation types, and data modality. The kinship dataset establishment has its specific characteristics. We will discuss the features of kinship datasets and their main issues.

(1) *Difficulty of collecting kinship datasets* Kinship datasets are based on pair-wise or group-wise samples. This causes an increased workload in data collection, annotation, and computation.

In addition, establishing a kinship dataset requires obtaining subjects’ private family-related information and family members’ images. Due to privacy protection laws (*e.g., *General Data Protection Regulation (GDPR) in Europe Union^5^), family information is not easy to be obtained and made public. Therefore, the existing kinship datasets are still small and not diverse enough.

(2) * Date diversity* Facial images in existing kinship datasets are usually captured with the camera straight to the subject. Typical conditions of important applications are barely considered, such as public surveillance cameras (*e.g., *finding missing children by using the surveillance network, tiny face kinship verification), identification photos (*e.g., *cross-domain kinship verification), and occlusion kinship verification (*e.g., *during COVID-19 period Goyal and Meenpal, 2021). Additional modalities (*e.g., *3D face modeling Crispim et al., 2020, infrared images, gait Bekhouche et al., 2020, expressions Dibeklioğlu et al., 2012a) should be considered and proven helpful in solving kinship verification under particular conditions. Moreover, exceptional cases where parents are not strictly from the same ethnicity (*e.g., *Melanoderm, and Caucasian) are barely carefully considered so far.

(3) * Cross-disciplinary dataset* Establishing a kinship dataset that includes DNA profiles could promote the cross-disciplinary study on how single nucleotide polymorphisms (SNP) affect facial heritage patterns (Schneider et al., 2019). As indeed, SNP allele demonstrates geographical or ethnic group particularities in facial appearance.^6^ From forensics perspective, the use of kinship matching and SNPs in phenotypic clues (*e.g., *hair color, eye color) inference on facial reconstructions can be applied to search suspects (Kayser, 2015).

## Kinship Verification from Still Images

Facial kinship verification from still images is popular, mainly due to the easily obtainable datasets and its wide range of applications. Generally, the kinship datasets contain the pre-processed facial images. Facial images are cropped and resized into a normalized size. Main efforts are dedicated to kin feature extraction and distance measurement. Then classifier is utilized for binary classification.

The remainder of this section is organized as follows. In Sect. 4.1, key steps of facial kinship verification are introduced briefly. In Sect. 4.2, traditional kinship verification methods are reviewed from feature learning (Sect. 4.2.1) and metric learning (Sect. 4.2.2) aspects. In Sect. 4.3, we introduce the recently emerged deep learning methods. In the end, Sect. 4.4 summaries this section and discusses the open issues.

### The Key Steps for Facial Kinship Verification

(1) *Face detection, alignment and segmentation* The goal of this step is to do face detection based on the input raw facial images. After locating the face, the eyes’ position is usually taken as the key feature to align the face. The purpose of face alignment and face adjustment is to reduce the influence brought by face scale and angle. The commonly used methods for face segmentation and alignment include MTCNN (Zhang et al., 2016) and ERT (Kazemi and Sullivan, 2014). Extensive research reviews on this sub-task have been carried out, for example, the survey work of Wu and Ji (2019).

(2) * Kin feature extraction* The two input facial images can be represented as $\mathbf {X}$, $\mathbf {Y}$. We extract features for these two facial images and denote them with vectors, $\mathbf {x}$, $\mathbf {y}$. Then kin features will be employed for the distance measurement and classification in the next step. The kin feature extraction step is an important research topic, and it also affects how the performance will be. Before deep learning techniques are used in kinship verification, some common handcrafted descriptors are applied. With the implementation of deep learning in kinship verification problems, the traditional feature descriptors are replaced by deep embeddings gradually. We will review the traditional representative methods and deep learning methods in Sects. 4.2.1 and 4.3.

(3) * Distance measurement* By extracting facial image features, two inputs are represented as two vectors. Then a proper distance metric is used to calculate the distance of two inputs in the feature space and assess the similarity between two faces. Metric learning aims to learn a transform matrix to narrow the distance between kin pairs (positive pairs) and enlarge the distance between non-kin pairs (negative pairs). The extracted facial features can be mapped into a new feature space and improve the performance of kinship verification (Lu et al., 2014c; Yan et al., 2014a, b). We will review the related metric-based methods in Sect. 4.2.2

(4) * Classification* The steps above produce a distance value between sample pairs. Kinship verification is a binary classification problem where commonly used classifiers are K-Nearest Neighbor (KNN), Support Vector Machine (SVM), and threshold classification.

### Traditional Methods

As kinship verification is a relatively new and challenging problem, many kinship verification methods were proposed during the last decades. At the beginning of the kinship verification research, traditional methods were proposed for solving the kinship verification problem. They showed good verification performance with computational efficiency, especially in small datasets. In this subsection, we review the traditional methods from aspects of feature extraction and metric measurement.

#### Feature Extraction Methods

**Fig. 6:**
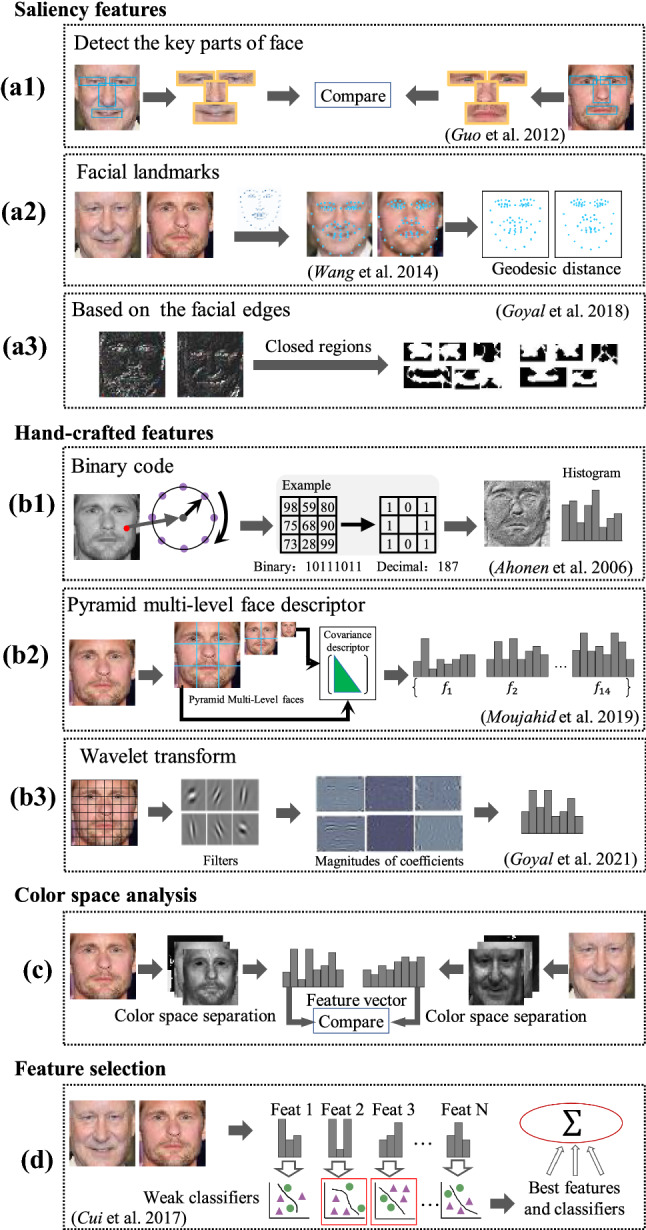
The illustration of facial kinship verification from traditional feature learning methods. Saliency feature-based methods include **a1** utilizing key facial parts (Guo and Wang, 2012), **a2** detecting facial landmarks (Wang and Kambhamettu, 2014) and **a3** learning facial features by closed edge regions (Goyal and Meenpal, 2018). Hand-crafted feature representations include **b1** LBP descriptors (Ahonen et al., 2006), **b2** proposed pyramid facial descriptors with learning covariance attributes between different facial patches (Moujahid and Dornaika, 2019) and **b3** wavelet transform (Goyal and Meenpal, 2020). In **c**, features combining color information methods can be sort into pre-defined color space-based  (Wu et al., 2016a) and learned color space (Liu et al., 2016). In the end **d**, the feature selection method aims to seek efficient ones among multiple facial features (Cui and Ma, 2017)

To establish an automatic facial kinship verification system, we first need to represent the faces with features effectively. We categorized these methods into enumeration features, facial saliency features, hand-crafted features, feature transformation based on color spaces, and feature selection methods, as shown in Fig. 6. Naive enumeration features started with the work of Fang et al. (2010b), which represented the facial traits from low-level features with different points of view, such as eye color, skin color, hair color, geometric characteristics between facial key points (eye, mouth, nose) and face shapes (size of the eyes, mouth or nose). Later, (Xia et al., 2012a, b) included more descriptive information, such as age, gender, and race. These features are represented with binary features encoded as $-1$ and $+1$. Nevertheless, the enumeration of these features needs manual efforts to label the samples, while the resulting features are usually low-dimensional and not comprehensive enough.

(1) * Kinship verification based on saliency features* Methods of kinship verification based on saliency aim to verify kinship by comparing the similarity of salient facial parts, such as nose, eyes, mouth (Goyal and Meenpal, 2018; Guo and Wang, 2012; Kohli et al., 2012; Wang and Kambhamettu, 2014). Thus, we need to first locate the facial key points. Given a facial image, to find the salient parts,  (Guo and Wang, 2012) proposed to utilize the eyes, mouth, and nose as the salient facial area. DAISY descriptor (Tola et al., 2009) is applied to extract features and compute the similarity between the image pairs. Kohli et al. (2012) proposed the Differences of Gaussians (DoG) method to locate the facial key parts. Then in 2014, Wang and Kambhamettu (2014) introduced the widely used 68 facial landmarks Asthana et al. (2013) extracted from facial images into kinship verification. Besides the methods that extract facial key points and facial landmarks, Goyal and Meenpal (2018) proposed an edge detection-based kinship feature extraction method. The Canny operator (Canny, 1986) was used for detecting the facial edges, and areas enclosed by them were considered as salient parts.

(2) * Hand-crafted features* The previous subsections introduced methods based on the facial shape. These methods are usually affected by detection accuracy, facial expression variance, noise, and face rotation, resulting in low verification accuracy and low noise tolerance under complex conditions. To solve these problems, researchers proposed feature descriptor methods (Goyal and Meenpal, 2020; Laiadi et al., 2021; Moujahid and Dornaika, 2019; Patel et al., 2017; Puthenputhussery et al., 2016; Yan, 2019; Zhou et al., 2011, 2012) for kinship verification. Among them, Local Binary Pattern (LBP) (Ahonen et al., 2006) is a widely used hand-crafted feature extraction method. LBP is an operator that describes the image’s local texture information. The resulting binary code describes the texture characteristics of an image block and is invariant to both rotation and gray-scale conversion (Ojala et al., 1996).

Based on the basic hand-crafted features, many methods improve the performance in different ways. Pyramid Multi-level covariance descriptor (PML-COV) (Moujahid and Dornaika, 2019) combined the LBP and HOG features extracted from multiple resolutions to establish the feature pyramid. Goyal and Meenpal (2020) proposed the Selective Patch-based Dual-Tree Complex Wavelet Transform (SP-DTCWT) method that decomposes the facial image using six wavelet functions. By computing the similarity between corresponding patches of an image pair, they can get discriminative feature patches for kinship verification.

(3) * Color texture transform-based methods* The traditional hand-crafted features are extracted from gray-scale images, thus ignore the useful chrominance features. The chrominance of facial images contains kinship heredity information, such as eyes’ color, skin color, hair color, etc. To make full use of color information from facial images, Wu et al. (2016a) proposed a color-texture feature extraction method to combine color features with texture features for kinship verification, making the features more discriminative. The proposed method first transforms the image into the targeted color space and then extracts features from each color channel. Experimental results demonstrated that the HSV color space can provide more abundant kinship information compared with other color spaces. Other related studies also indicate the effectiveness of color information to solve the kinship verification problem (Laiadi et al., 2019; Wu et al., 2016b).

Besides extracting color features from the existing color spaces, (Liu et al., 2015, 2016) proposed an Inheritable Color Space (InCS) that views the kinship distance metric as the objective function to learn an inheritable transformation $\mathbf {W}$. $\mathbf {W}$ can map the images from the original color space to the new color space. Upon the two kin images $\mathbf {X}$ and $\mathbf {Y}\in \mathbb {R}^{3\times n}(n=h\times w)$, the images are transformed to a new color space, $\hat{\mathbf {X}}=\mathbf {W}^T\mathbf {X}$, $\hat{\mathbf {Y}}=\mathbf {W}^T\mathbf {Y}$, where the distances between kin pairs are closer than non-kin pairs.The components of learned color spaces are decorrelated and have low information redundancy. Besides that, the InCS is robust to the illumination variations. Experimental results on multiple datasets have shown the superiority of InCS compared to other color spaces.

(4) * Feature selection* Unlike single feature extraction methods, feature selection aims to study fusion schemes by selecting among multiple features, enriching feature representations, and reducing feature redundancy (Alirezazadeh et al., 2015; Bottinok et al., 2015; Chen et al., 2017; Cui and Ma, 2017). Usually, the inputs of feature selection methods are multiple feature representations. They can select the most effective representations by introducing a constraint as an objective function or directly as the classification accuracy. Alirezazadeh et al. (2015) first proposed to fuse local and global features and select the valuable and discriminative features for kinship verification. Bottinok et al. (2015) extracted multiple features from images, including Local Phase Quantization (LPQ), Weber’s Local Descriptor (WLD), and LBP. Before they classify the features, to improve the verification accuracy, they propose the Max-Relevance and Min-Redundancy (mRMR) method to select a subset of variables to best describe the data.

Beyond that, researchers also use the classifier as a guide for feature selection (Fig. 6d). Cui and Ma (2017) proposed an adaptive feature selection method. They used a matrix $\mathbf {W}$ to select discriminative features. For one given feature kind $\mathbf {f}_j,j=1,\dots ,N$, they trained a weak classifier $\mathbf {h}_j,j=1,\dots ,N$. *N* weak classifiers selection and optimization can be achieved through an objective function as follows.

2 $$\begin{aligned} \begin{aligned} \min _{\xi ,{\varvec{\omega }}_j,\mathbf {b}_j}\left\| {\varvec{\omega }}_j \right\| ^2&+C\sum _{i:y_i=1}^{N_t} W_i\xi _i\\ s.t.\quad y_i({\varvec{\omega }}_j^T\Phi (\mathbf {x}_{ij})+\mathbf {b}_j) \ge&1-\xi _i,i=1,\cdots ,N\\ \xi _i \ge 0,i&=1,\cdots ,N \end{aligned} \end{aligned}$$

Where, $W_i$ is the regularization parameter, $\xi _i$ is the slack variable for each sample pair, ${\varvec{\omega }}_j,\mathbf {b}_j$ are the hyper-parameters of *i*th SVM, *C* is the trade-off parameter, $\mathbf {x}_{ij}$ is the difference of *i*th sample pair with respect to the *j*th feature representation, $\Phi (\mathbf {x}_{ij})$ is the feature map for the input space. By optimizing this objective function, multiple weak classifiers are elected to construct the final strong classifier. Similar to (Chen et al., 2017; Cui and Ma, 2017) applied Canonical Correlation Analysis (CCA) to find a multiple feature mapping function to improve the correlation of kin pairs.

(5) * Other methods* Besides the methods introduced above, researchers also tried to solve the feature extraction from other points of view. Fang et al. (2013b) selected multiple facial parts from different people to construct a part-based dictionary. The features of a queried facial image can be reconstructed by sparse coding using this dictionary. Chen et al. (2020) used the dictionary learning method to reduce the gap between kin facial images. Duan and Tan (2015) proposed a feature subtraction method to remove the unrelated kinship part from the local feature and retain valuable information. Bessaoudi et al. (2019) extracted the high-order representations of facial features. And (Laiadi et al., 2020) used Tensor Cross-view Quadratic Discriminant Analysis (TXQDA) method. They use the feature mapping method to learn low-dimension tensors to reduce the factors brought by age and gender.

**Fig. 7:**
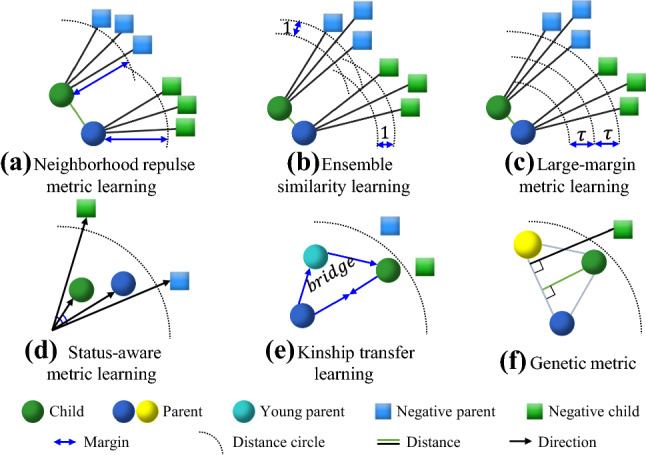
Illustrations of metric learning methods for kinship verification. Circles in the figure represent kin nodes, and squares with the corresponding color are negative kin samples. The dashed lines are radii that represent distance margins. **a** illustrates the NRML method (Lu et al., 2014a) that repulses non-kin images within node’s own neighbor circle. **b** Ensemble similarity learning (Zhou et al., 2016a) is similar to NRML, while it enlarges the neighbor circle with an additional constant. **c** Large-margin metric learning (Hu et al., 2017) takes all positive and negative pairs together and introduces a large margin to separate negative samples. **d** State-aware metric learning (Liu and Zhu, 2017) computes angle between two features. **e** Transfer learning method (Shao et al., 2011a) takes young parent as a bridge to learn a mapping function, thus to pull kin pairs with age gap closer. **f** Genetic metric (Zhang et al., 2016) obtains the intrinsic distance from child to both parents in an unsupervised way

#### Metric Learning Methods

Metric learning was firstly proposed by Eric Xing et al. (2002) on NIPS 2002. For the kinship verification problem, we would need to find a proper distance measurement method to compute the distance between an image pair based on feature extraction methods. Ideally, in this metric, the image pairs with kin relations (positive pairs) would have small distances, while those without kin relations (negative pairs) would have large distances. It maps the distance metric space into a new metric space (Kulis, 2012). The commonly used basic distance metrics in kinship verification are Euclidean distance (Yan et al., 2014b), Mahalanobis distance (Hu et al., 2014, 2017; Kou et al., 2015; Lu et al., 2014c; Wei et al., 2019; Yan et al., 2014a; Zhang et al., 2015), bilinear similarity (Fang et al., 2016; Qin et al., 2016; Xu and Shang, 2016a, b; Zhou et al., 2016a, b), graph learning (Guo et al., 2014; Liang et al., 2018), cosine similarity (Yan et al., 2015; Yan, 2017), CCA (Lei et al., 2017) and other metric patterns (Liu et al., 2017; Liu and Zhu, 2017; Wu et al., 2018; Zhang et al., 2016; Zhao et al., 2018). Selective methods are illustrated in Fig. 7. We will review these methods according to the metric categories.

(1) * Neighborhood Repulsed Metric Learning* In 2014, Lu et al. (2014c) proposed the Neighborhood Repulsed Metric Learning (NRML) method for kinship verification, which is also the first try of metric learning in solving kinship verification, and provided the fundamental theory and protocol for metric learning-based kinship verification study. The motivation of NRML is that the negative neighbors of positive samples can confuse the classifier. Based on that, NRML repulses the *k* negative neighbors and pulls the positive samples together, thus separating the positive samples and negative samples (Fig. 7a). The training set is represented as $S=\left\{ \left( \mathbf {x}_i,\mathbf {y}_i \right) \mid i=1,2,\cdots N \right\}$, where there are *N* kin image pairs. The distance between $\mathbf {x}_i$ and $\mathbf {y}_j$ is computed with Mahalanobis distance, $d(\mathbf {x}_i,\mathbf {y}_j)=\sqrt{(\mathbf {x}_i-\mathbf {y}_j)^T\mathbf {W}(\mathbf {x}_i-\mathbf {y}_j)}$. The $\mathbf {W}$ is a symmetric and positive semidefinite matrix. The objective of NRML is to seek a proper $\mathbf {W}$ to achieve that when $i=j$ the distance between $\mathbf {x}_i$ and $\mathbf {y}_j$ is as small as possible, otherwise the distance should be as large as possible. The objective function is denoted as,

3 $$\begin{aligned} \begin{aligned} \max _{\mathbf {W}} J(\mathbf {W})&=J_{1}(\mathbf {W})+J_{2}(\mathbf {W})-J_{3}(\mathbf {W}) \\&=\frac{1}{N k} \sum _{i=1}^{N} \sum _{t_{1}=1}^{k} d^{2}\left( \mathbf {x}_{i}, \mathbf {y}_{i t_{1}}\right) \\&\quad +\frac{1}{N k} \sum _{i=1}^{N} \sum _{t_{2}=1}^{k} d^{2}\left( \mathbf {x}_{i t_{2}}, \mathbf {y}_{i}\right) -\frac{1}{N} \sum _{i=1}^{N} d^{2}\left( \mathbf {x}_{i}, \mathbf {y}_{i}\right) \\ \end{aligned} \end{aligned}$$

where, $\mathbf {y}_{i t_{1}}$ denotes as the $t_{1}$th sample in *k* nearest negative neighbors of $\mathbf {y}_i$, $\mathbf {x}_{i t_{2}}$ denotes as the $t_{2}$th sample in *k* nearest negative neighbors of $\mathbf {x}_i$. The first two terms of Eq. 3 aims to repulse the negative samples of $\mathbf {x}_i$ and $\mathbf {y}_i$ within *k* nearest neighbors. While $J_{3}(\mathbf {W})$ pushes the positive samples $\mathbf {x}_i$ and $\mathbf {y}_i$ together. Thus NRML algorithm can set positive and negative samples apart. $\mathbf {W}$ is solved by iteratively updating the variables.

NRML method showed the best performance at that stage in 2014, achieving $73.8\%$ and $69.9\%$ verification accuracy on KinFaceW-I and KinFaceW-II datasets. The main idea of NRML is also used in other metric learning methods. Yan et al. (2014b) proposed to map the feature vectors into the hyperplane of SVM and applied the NRML method to optimize the distance metric. Xu and Shang (2016a) concatenated multiple features into one vector and combine the NRML method with bilinear similarity to compute the distance between image pairs. Yan et al. (2015) and Lei et al. (2017) replaced the distance metric with cosine similarity and CCA. They also demonstrated the effectiveness of NRML.

Besides the reviewed NRML related methods, researchers also proposed other metric learning methods based on Mahalanobis distance. Yan et al. (2014a) introduced a probability model, where the probability of positive pairs having a smaller distance than most similar negative pairs is maximized, which can be formulated as $P(d(\mathbf {x}_i,\mathbf {y}_i)<d(\mathbf {x}_i,\mathbf {y}_j))$. By minimizing *P*, $\mathbf {W}$ can be optimized simultaneously. Hu et al. (2014, 2017) proposed the large-margin multi-metric learning (LM3L, Fig. 7c) method to learn a metric based on fusing multi-view features. To further separate the positive and negative pairs, they introduced an extra margin.

(2) * Metric learning based on bilinear similarity* Besides the commonly used Mahalanobis distance measurement, bilinear similarity $S_{\mathbf {W}}\left( \mathbf {x}_{i}, \mathbf {y}_{i}\right) =\mathbf {x}_{i}^{T} \mathbf {W} \mathbf {y}_{i}$ is also used for the metric learning-based kinship verification studies, where $\mathbf {W}$ is the positive semidefinite matrix. When $\mathbf {W}$ is the identity matrix, bilinear similarity can be viewed as the cosine similarity without normalization. Bilinear similarity has shown good performance for image retrieval (Deng et al., 2011; Gao et al., 2014) and it can effectively calculate the similarity between two sparse feature vectors.

Zhou et al. (2016a, b) proposed the Ensemble Similarity Learning (ESL) method (shown in Fig. 7 (b)) to solve the kinship verification problem. The inputs are $\mathbf {x}_{i}, \mathbf {y}_{i}, \mathbf {x}_{j}, \mathbf {y}_{j}$. The objective function based on their distance are,

4 $$\begin{aligned} \left\{ \begin{array}{l} S_{\mathbf {W}}\left( \mathbf {x}_{i}, \mathbf {y}_{i}\right) \ge S_{\mathbf {W}}\left( \mathbf {x}_{i}, \mathbf {y}_{j}\right) +1 \\ S_{\mathbf {W}}\left( \mathbf {x}_{i}, \mathbf {y}_{i}\right) \ge S_{\mathbf {W}}\left( \mathbf {x}_{j}, \mathbf {y}_{i}\right) +1 \end{array}\right. \end{aligned}$$

Where, $\mathbf {x}_{i}$ and $\mathbf {y}_{i}$ represent the positive pairs. $\mathbf {x}_{j}$ and $\mathbf {y}_{j}$ represent negative samples.

ESL method has superior computational efficiency and can be applied for high-dimensional data. Then the inputs of ESL are quadratic, which satisfies the inter- and intra- constraints on the similarity pattern for image pairs. Qin et al. (2016) proposed a multitask-based bilinear similarity learning method. They combined the four kinship verification tasks to transfer the knowledge from one task to other tasks. Fang et al. (2016) introduced the logistic loss to smooth the objective function and improve the efficiency of the optimization process.

(3) *Other metric learning methods* Besides the metric learning reviewed above, researchers also proposed methods from other points of view. Zhang et al. (2016) proposed a generic metric. In the feature space, the distance between a child and two parents can be computed by the minimum length from the child feature vector to the feature vectors of two parents. Liu et al. (2017, 2017) introduced the angle $\theta$ between the parent’s and child’s feature vector to formulate the objective function. Wu et al. (2018) introduced a low-rank metric learning method to learn the latent subspace and dig more discriminative representations adaptively. Zhao et al. (2018) proposed the multi-kernel metric learning method, including linear and nonlinear distance metric methods. By weighted fusing them, they can obtain the final distance. The graph learning method is also studied for metric learning-based kinship verification. Liang et al. (2018) build the Intrinsic Graph and Penalty Graph according to the relationship between the data. They combined the NRML algorithm and graph learning to describe the intraclass compactness and interclass separability.

Age variance between parents and children can have an adverse effect on kinship verification. Shao et al. (2011a), Xia et al. (2011) pointed that children and their parents look more alike when parents are at young ages. The idea of reducing the divergence caused by the aging effect is to utilize the young parent’s facial images as a bridge between children and elder parents. The module takes images of young parents, old parents, and children as the source, intermediate, and target, which can be denoted as $\mathbf {X_{yp}}$, $\mathbf {X_{op}}$ and $\mathbf {Y}$. A subspace projector matrix $\mathbf {W}$ is learned to project the intermediate domain and the other two domains to have the same distribution. One drawback of this study is that it requires manual efforts to collect the images of parents both when they are old and young.

Metric learning methods project the feature vectors into a new feature space that pulls the kin image pairs together and pushes the non-kin image pairs further away. In this subsection, we reviewed and summarized the existing metric learning-based kinship verification methods. Traditional metric learning methods are based on the feature extraction module. Besides that, deep metric learning methods integrate the feature extraction and metric learning loss to guide the deep network to learn comprehensive feature extraction strategies. We will review these methods in the following subsection.

### Deep Learning Methods

Traditional hand-crafted feature extraction methods have limited ability on feature description. While the CNN-based deep learning methods have a strong capability of non-linear expression. They can learn the effective feature embeddings from the original raw data by applying task-related constraints, thus avoiding the traditional hand-craft feature extraction rules (Ma et al., 2021; Xia et al., 2020a, b).

**Fig. 8:**
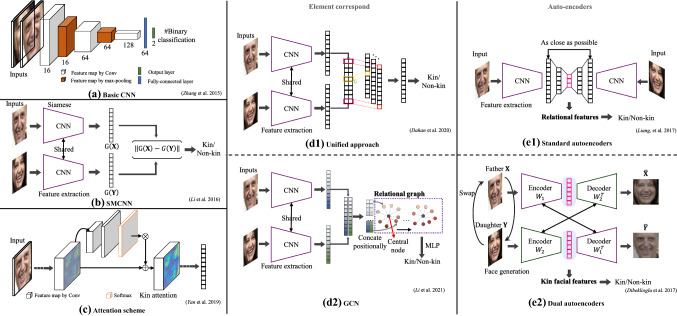
Deep learning-based kinship verification methods. **a** Basic CNN-based (Zhang et al., 2015). **b** Deep metric learning method SMCNN (Li et al., 2016) with Siamese architecture. **c** Attention scheme (Yan and Wang, 2019) in kinship verification. **d1** and **d2** are approaches that aim to analyze corresponding embedding elements of two facial images. **d1** Unified approach (Dahan and Keller, 2020) uses row convolution, and **d1** GNN (Li et al., 2021a) introduces GCN to build a relational graph. **e1** and **e2** are architectures based on auto-encoders. **e1** applies single stream autoencoders to learn the relational features of two facial images (Liang et al., 2017). **e2** illustrate a dual autoencoders architecture with each stream leaning kin features (Dibeklioglu, 2017)

With the fast development of deep learning in computer vision and the emergence of large-scale kinship datasets, researchers started to study the deep learning methods for kinship analysis in 2016 (Li et al., 2016). The existing facial kinship verification algorithms have used multiple novel deep architectures, including basic neural networks (Wang et al., 2015), deep metric learning (Li et al., 2016), architectures based on auto-encoders (Gao et al., 2019; Kohli et al., 2019b; Wang et al., 2015) and attention networks (Yan and Wang, 2019), etc, as shown in Fig. 8. We will summarize and review these methods in the latter part of this section.

The very first method proposed by Wang et al. (2015) in 2015 has two stages: feature extraction and deep metric learning. The facial features are extracted with traditional methods. Features are fed into nonlinear AutoEncoders followed with Mahalanobis distance metric to project the features into a non-linear space. The drawback of the method is that the input is the LBP feature, and the detailed information of the original image is missing. The first End-to-End deep learning method for kinship verification is proposed by Zhang et al. (2015). The architecture is shown in Fig. 8a. The network inputs are two stacked facial images, and then outputs the final result. The architecture of the network is simple yet effective.

(1) * Deep metric learning methods* To optimize the distance between two input facial images, researchers proposed to add a distance metric into network training, which we call Deep Metric Learning methods (Duan and Zhang, 2017; Li et al., 2016, 2017; Lu et al., 2017; Zhang et al., 2020; Zhou et al., 2019). The typical network architecture is Siamese Network, which is shown in Fig. 8b. Different from one-stream networks, Siamese networks have two streams that share the same weights and utilize the distance metric as the loss function to learn an optimal feature space such that positive pairs (pairs with kin relation) have small distances and negative pairs (pairs without kin relation) have large distance.

Li et al. (2016) proposed the Similarity Metric based Convolutional Neural Networks (SMCNN) method. The inputs of the network are two facial images, $\mathbf {X}$ and $\mathbf {Y}$. $G(\cdot )$ indicates the FC layer output of the network. They employed the $l_1$-norm to compute the distance of two output embeddings. Equation 5 formulates the distance between two embeddings.

5 $$\begin{aligned} D\left( \mathbf {X},\mathbf {Y} \right) =\left\| G\left( \mathbf {X} \right) -G\left( \mathbf {Y} \right) \right\| _1 \end{aligned}$$

During the training, Li *et al*. added a threshold $\tau$ to further partition the positive samples and negative samples. The labels of positive samples and negative are denoted as $y=1$ and $y=-1$. Then we can have the cost function of the network.

6 $$\begin{aligned} L_{SMCNN}=f(1-y(\tau -D(\mathbf {X},\mathbf {Y}))) \end{aligned}$$

where $f(\cdot )$ is the generalized logistic loss. To minimize the cost function, the gradient descent algorithm is adopted to optimize the convolutional neural networks.

Moreover, the commonly used metric-based loss functions include Contrastive Loss and Triplet loss (Dibeklioglu, 2017; Wu et al., 2019). These two loss functions are based on distance measurement, such as Euclidean distance. The contrastive loss takes positive pairs and negative pairs as inputs. Different from contrastive loss, triplet loss has three inputs, including the Anchor (a), the Positive (p), and the Negative (n). The positive and negative pairs are referring to the anchor sample. Thus, positive sample pairs are clustered, and the positive and negative samples are separated.

While regarding the deep metric learning techniques, the selection of the sample pairs/tuples can directly affect the efficiency and performance of the network. Researchers proposed the Hard Sample Mining methods (Li et al., 2021b; Suh et al., 2019; Wang and Yan, 2020). Hard sample mining methods are designed to find positive sample pairs with large distances and negative sample pairs with small distances from training batches, which can produce large backward losses and effectively train the network. Li et al. (2021b) proposed a discriminative sample mining approach using meta-learning in kinship verification. They abandoned the easy negative ones and kept the hard samples to dominate the gradient.

(2) * Architectures based on auto-encoders* Another deep kinship verification architecture is based on Auto-Encoders (AE) (Dehghan et al., 2014; Dibeklioglu, 2017; Ertugrul and Dibeklioglu, 2017; Gao et al., 2019; Kohli et al., 2019b; Liang et al., 2017; Wang et al., 2015, 2016, 2017). The very first applied autoencoders in kinship verification aims to train a model for facial feature extraction (Wang et al., 2015). The encoded feature is the reduced feature representation of the input.

Many auto-encoder methods were motivated by the correlation between inputs and outputs. They can be categorized into two classes, traditional autoencoder (Kohli et al., 2019b; Wang et al., 2016, 2017) and NN-based autoencoder (Dehghan et al., 2014; Dibeklioglu, 2017; Liang et al., 2017). Traditional autoencoder learns the relation mapping representation by minimizing the loss function formulated to fit two input images. The NN-based autoencoders use multiple layers of projection and optimize the network by back-propagation. Liang et al. (2017) proposed to utilize the intermediate layer to describe the relationship between inputs and outputs. They first extracted the features of two facial images by a pre-trained CNN. The obtained features are the inputs of autoencoders. By minimizing the difference between the encoded feature and the child’s feature, the autoencoders can be optimized, and the output of the intermediate layer shows the relational feature of the two facial features, as shown in Fig. 8e1. The method proposed by Liang *et al*. requires learning the rational feature every time when new input pair comes.

Dibeklioglu (2017) improved it by encoding both inputs into a dual network and defined comprehensive losses to learn kin-related features in an End-to-End fashion. Though they utilize the video frame as the input that should be reviewed in the Sect. 5, we prefer to put it here for the consideration of completion. They took a pair of kin images as the inputs of dual autoencoders, as shown in Fig. 8e2. They made the output of each decoder similar not only to the input facial image but also to its kin facial image. At last, they adopted the encoded features as the kin feature representations. Formally, two input images $\mathbf {X}$ and $\mathbf {Y}$ pass through the autoencoders and then two generated facial images $\hat{\mathbf {X}}$ and $\hat{\mathbf {Y}}$ are obtained. The kinship loss is defined to maximizing the similarity of kin pairs as follows:

7 $$\begin{aligned} L_{kin}=D(\mathbf {{X} },{\hat{\mathbf {Y}}})+D(\mathbf {{Y}},{\hat{\mathbf {X}}}) \end{aligned}$$

Where $D(\cdot ,\cdot )$ denotes the distance between two images. Kinship loss aims to learn the facial transformation between kin pairs and maintains the effective kin feature representation. The non-kinship loss aims to minimize the similarity of non-kin pairs. $\mathbf {X}_j$ and $\mathbf {Y}_j$ are negative samples with regards to the input parent’s image and child’s image, respectively. Then the non-kinship loss is formulated as:

8 $$\begin{aligned} L_{non-kin}=-D({\hat{\mathbf {Y}}},\mathbf {X}_j)-D({\hat{\mathbf {X}}},\mathbf {Y}_j) \end{aligned}$$

Combining both kinship loss and non-kinship loss, the final loss can be written as:

9 $$\begin{aligned} L_{ae}=\lambda L_{kin}+(1-\lambda )L_{non-kin} \end{aligned}$$

where $\lambda$ is a tradeoff parameter between kinship loss and non-kinship loss. By training the auto-encoders, the distance of encoded features indicates the relationship of the inputs.

Moreover, some researchers applied image synthesis and generative techniques to synthesis a child’s facial images given parent’s facial image by Generative Adversarial Networks (GANs) (Gao et al., 2019; Ozkan and Ozkan, 2018). Besides, GANs can also be utilized to learn disentangled images or representations when facing challenges such as age and gender. Wang et al. (2018) applied GANs as a cross-generation framework towards generating young parents. The old parents were transformed to their young ages to mitigate the age gap. To mitigate the gender difference, Feng and Ma (2021) proposed Gender-FEIT. GANs were trained to learn gender invariant face representation in the case of the opposite gender (*e.g., *FD, MS). A gender discriminator was imposed on the encoder to train the network in an adversarial manner.

(3) * Architectures based on attention scheme* The psychological research indicates that kin clues are located in the specific areas of the face rather than in the entire face (Dal Martello and Maloney, 2006). Methods discussed above take the whole face as a clue for verifying kinship while ignoring the facial kin feature distribution. In order to learn an effective kin feature embedder, multiple attention mechanisms can be applied to guide the network to pay attention to genetic regions. One possible and widely used method is the channel-wise attention mechanism (Zhang et al., 2021). It learns an adapting weight for different feature channels, as it is assumed that channel-wise features reflect variant information over space. By training deep networks with kin-constrained loss function, the kinship-interested feature is generated.

The attention proposed in Yan and Wang (2019) is illustrated in Fig. 8c. Yan *et al*. learned the facial geometric weights directly from the transformation of the intermediate feature map. They also applied the residual learning idea to retain original information by summing the weighted feature map with the original feature map. Specifically, the feature map passes through a pooling operation and convolutional layer. To restore the feature map with the same size as the original feature map, they used an up-sampling method followed by a sigmoid function to map the weights into 0 to 1 scale. The original feature map is formulated as $C(\mathbf {X})$ and $F(\mathbf {X})$ denotes the attention weights. The weighted feature is denoted as $P({\mathbf {X}})=F(\mathbf {X}) *C(\mathbf {X})$. To avoid the loss of information, Yan *et al*.applied the residual method $P({\mathbf {X}})=(1+F(\mathbf {X}))*C(\mathbf {X})$. The attention network shows good performance on KinFaceW-I and KinFaceW-II datasets. They reached 82.6% and 92.0% accuracies, respectively, which are superior compared to basic CNN.

The attention networks learn the interest areas for the kinship verification task. While the study of that is still in its infancy, and only limited studies can be found. Researchers (DeBruine et al., 2009) also pointed that age and gender gaps influence the verification accuracy of kinship. Thus, locating the age and gender invariant regions containing kinship clues using the attention method is also promising in the future.

(4) * Other architectures* Besides the deep architectures we have reviewed above, researchers also contributed to solving other problems. Yan and Song (2020) studied the latent CNN embeddings for kinship verification. The two input facial images pass through weight-shared CNNs, and they collect the embeddings from different layers of CNNs. The embeddings are respectively concatenated to measure the kin similarity. Similar to Yan’s method, Dahan and Keller (2020) collect the embeddings from the last FC layer, and a fusion mechanism is proposed to learn the similarity and dissimilarity of input images.

Zhang et al. (2019) proposed an appearance and shape-based deep learning method. They extracted both appearance and shape features and combined them for kinship verification. Experimental results indicate that compared with single feature representation, fusing appearance and shape features can improve the verification accuracy by 10%. Li et al. (2020, 2021a) pointed that we usually inferred the kinship by comparing the corresponding facial attributes of two persons. In their approach, they took every dimension of a CNN output as one genetic feature. Based on that, they established a kin graph, where the node is represented with the concatenation of the corresponding *d*th bits obtained from two feature vectors $g(\mathbf {x})$ and $g(\mathbf {y})$.

Kinship verification studies suffer from insufficient training data, especially for the efficient training of deep learning models. To solve this problem, Song and Yan (2020) proposed a kinship data augmentation method named KIN-MIX, which augments data from the feature level rather than raw facial images. A linear sampling method was used to generate positive kin samples by mixing a pair of kin features. The generated feature can be represented as $\mathbf {z}=\lambda \mathbf {x} + (1-\lambda )\mathbf {y}$, where $\mathbf {x}$ and $\mathbf {y}$ are features with kinship, $\lambda \in [0,1]$, and $\mathbf {z}$ is the augmented feature. Their experimental results indicated that there is a performance improvement when training with the augmented data.

### Summary and Discussion

Methods of FKV based on still images aim to obtain kinship discriminative features from facial images and apply the mathematical models to represent the resemblance between kinship. We have witnessed significant progress on various aspects of facial kinship verification. In this subsection, we will summarize the main achievement of FKV, discuss the main issues.

#### The Status of FKV

Over the last decade, image-based kinship verification techniques have been developed to a great extent. In the early research, first attempts demonstrated the possibility of automatically verifying kinship with computer vision methods. With machine learning methods showing great potential, kinship verification got increasing attention in the field. Advances were made with many methods proposed. Brand new ideas of problem formulation (Lu et al., 2014c; Wang et al., 2015; Zhang et al., 2015), algorithms from different disciplines (Yan and Wang, 2019) were raised. More recently, deep learning methods (Dahan and Keller, 2020; Li et al., 2021b) have emerged and shown powerful learning capability on large datasets. Different methods showed different levels of progress towards the specific tasks. We will discuss the performance comparison in detail in Sect. 6.

#### Main Issues and Facts

(1) *Feature descriptor* For facial kinship verification problem, how to automatically locate genetic features from faces (*e.g., *by combining bio-genetic evidence) is remaining as a challenge. Besides, developing accurate and robust kin feature extraction methods is desired under complex environment and is essential to establish a robust kinship verification system.

(2) * Proper distance metric* The inter-class similarity and intra-class dissimilarity cause the kinship verification problem less discriminative. The optimal distance metrics or classifiers are critical for kinship verification. This is evidenced by methods, *e.g., *NRML (Lu et al., 2014a) and CNN-based methods (Dahan and Keller, 2020; Li et al., 2016, 2020), that kin and non-kin pairs are further separated by optimized metrics.

(3) * Reliability of training data* Kinship verification is achieved by comparing the similarity of two facial images (Lu et al., 2014a). Same photo problem (Lu et al., 2014c) were pointed out by Bordallo (Bordallo López et al., 2016) that FKV system would focus on unwanted clues. FKV system trained with the same photo data could be fragile when test kin faces are from different photos.

(4) * Family generalization* In FKV study, the training set has no family overlap with the testing set, which means that test subjects are unseen during the training. The model confronts with generalization problems when new family comes. Methodologies of Domain Generalization (Wang et al., 2021) (DG), *e.g., *domain-invariant representation learning and meta-learning, are potential in studying family-invariant heritage features.

(5) * Same person issue* As we have discussed in Sect. 2.1, it is reasonable that FKV system will give high prediction accuracy for images from same person. In specific scenarios (*e.g., *visa fraud 7), an image from the same person can easily spoof the system. The additional upper boundary for FKV is demanded.

## Kinship Verification from Facial Videos

Compared to still images, facial videos can provide more information. A video-based kinship verification system indicates the kin or non-kin relation between subjects present in video sequences containing faces. This is an important research problem for some use cases, such as surveillance systems or social media broadcasting. The first video-based kinship verification study dates back to 2013 (Dibeklioglu et al., 2013), when (Dibeklioglu et al., 2013) combined appearance and dynamic features to depict kin characteristics. Although video-based kinship verification is an extension of image-based kinship verification research, it contains additional spatio-temporal information that can be useful for FKV. However, due to the significant challenges we list below, video-based kinship verification has still not reached its full potential.

**Fig. 9:**
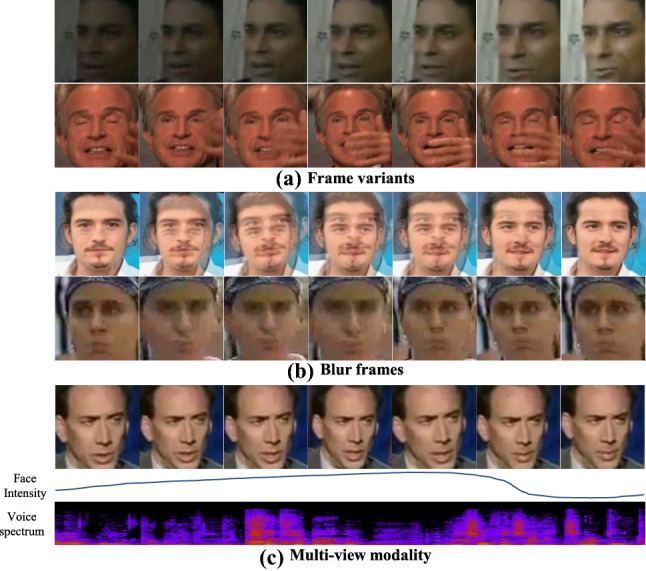
The challenges of video-based kinship verification

(1) * Low quality of facial videos* Typical facial videos are usually recorded with subjects that do not necessarily cooperate with the recorder. Hence, the facial quality shows more variability, especially in pose and illumination, which can fluctuate across subjects and frames of the same video. In addition, occlusion and target loss are also possible, as shown in Fig. 9a. Eliminating the noise while adaptively extracting helpful information is still an unsolved problem, which is usually mitigated in current datasets by simplifying the recording conditions (Dibeklioglu et al., 2013).

(2) * Blurry video frames* The understanding of moving faces in sequences is frequently hindered by frame blurring due to motion (see Fig. 9b). This is especially evident under slow shutter speeds and long exposure times (Shen et al., 2020). Advanced devices can address this issue by collecting data at higher frame rates, with high-quality optics and short exposure times. However, this can cause an unnecessary waste of resources (Jin et al., 2019). Deblurring video frames for kinship analysis still remains as a challenge.

(3) * Integration of faces, audio, and body information* Videos provide rich behavior information and dynamic cues besides facial appearance. Voice (Wu et al., 2019) and gait (Bekhouche et al., 2020) could act as complementary modalities that provide kin clues. The main challenge is to devise how to fuse multiple modalities properly to learn the complementary features for kinship verification.

Video-based kinship verification systems are similar to image-based kinship verification and follow a similar approach introduced in Sect. 4.1. The distinct difference is to model kin features from sequences. We review the existing video-based methods from constrained video-based kinship verification in Sect. 5.1 and unconstrained video-based kinship verification in Sect. 5.2. Sect. 5.3 summarizes the status of video-based kinship verification and discusses the remaining research issues.

### Constrained Video-Based Kinship Verification

Constrained video-based kinship verification refers to verifying kinship from facial videos that there is no variance on shooting environment and subject actions. A representative constrained dataset is the UVA-NEMO Smile dataset (Dibeklioglu et al., 2013) as we have discussed in Sect. 3. It is hypothesized that people with kin relations might also share similar facial expression dynamic features that could be present in *e.g., *smiling style. This hypothesis was corroborated by the original authors in 2013.

**Fig. 10:**
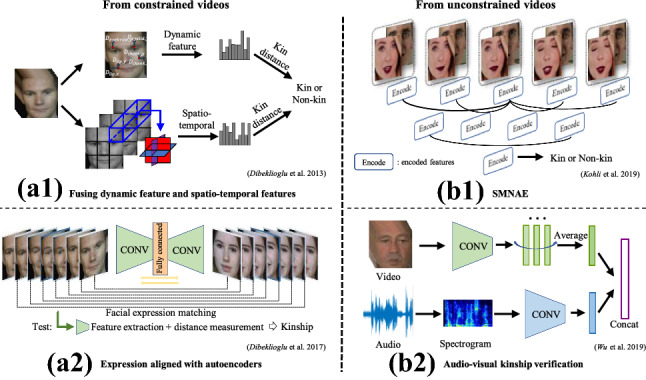
Illustration of video-based kinship verification methods. **a1** and **a2** methods based on constrained videos. **a1** Traditional methods fusing dynamic and spatio-temporal features (Dibeklioglu et al., 2013). **a2** learns kin features with matched smiling frames (Dibeklioglu, 2017). **b1** and **b2** methods are based on unconstrained videos. **b1** SMNAE (Kohli et al., 2019b) utilizes video frame pairs to learn comprehensive distance representation. **b2** Multi-modal method (Wu et al., 2019) fuses both facial and vocal features

Dibeklioglu et al. (2013) extracted the dynamic and facial spatio-temporal features for kinship verification. They localized 17 facial landmarks to track facial movement and extracted the dynamic features based on them. Together with the spatio-temporal feature CLBP-TOP, they demonstrated the family resemblance of smiling faces (Fig. 10a1). Boutellaa et al. (2017) combined deep features and spatio-temporal features (*e.g., *LBP-TOP) to study constrained video-based kinship verification. Experimental results showed that deep features have complementary information regarding spatio-temporal features. In 2017, Dibeklioglu (2017) proposed to measure the similarity of kin facial smile videos by matching affective intensity, as shown in Fig. 10 (a2). They decomposed the smile video into frames and aligned the sub-sequence according to the smile intensity of the face. The matched sequence pair is the input of dual auto-encoders, as reviewed in Sect. 4.3.

Constrained video-based kinship verification studies indicate that people with kinship have both similar appearance and smiling expressions. However, it requires strict collection conditions that hinder its applicability. To answer this limitation, researchers formulated unconstrained video-based kinship verification, which we will review in the following subsection.

### Unconstrained Video-Based Kinship Verification

Compared with constrained videos, unconstrained videos are collected in the wild conditions. Relaxing the restriction of the collection conditions makes it easier to enlarge the scale of the datasets. The collection of large video frames provides a larger number of individual frames to be used in training, but at the same time, it severely increases the burden of computation. On the other hand, the variability of the collected videos also provides for additional multimodal cues that could be exploited in a complementary manner. We review the methods from these two perspectives.

#### Frame-Wise Kinship Verification

Kohli et al. (2019b) proposed a three-stage autoencoder to learn the relation between two facial videos, called Supervised Mixed Norm AutoEncoder (SMNAE, as shown in Fig. 10b1). First, every video was decomposed into a sub-sequence with a specific number of frames, called *vidlet*. The vidlet pair is the input of the three-stage autoencoder. In the first stage, the relation of the corresponding video frame was learned as the facial resemblance. The second stage concatenated the spatio-temporal representations. In the end, the third stage fused the spatio-temporal information and learned the final score of kin probability. As introduced in Sect. 4.3, this method has a common drawback, since the learning procedure needs to be repeated for each input pair.

#### Multi-Modal Kinship Verification

Research works of Genetics (Sataloff, 1995), Psychology (Van et al., 2001) and Acoustics (Debruyne et al., 2002; Nolan et al., 2011; Weirich and Lancia, 2011; Whiteside and Rixon, 2013) indicated that speaking voice also had hereditary traits. In practice, this is intuitively known that we can recognize certain traits of the parent’s voice in their children’s speaking. Sataloff (1995) analyzed that related to the vocal function is genetically determined. Researchers in the field of acoustics have also demonstrated the voice similarity between kin-related people quantitatively (Debruyne et al., 2002; Nolan et al., 2011; Weirich and Lancia, 2011; Whiteside and Rixon, 2013). Besides that, psychology researchers found out that people have the ability to judge kinship only from voice (Van et al., 2001).

Multi-modal kinship verification was described by Wu et al. (2019) in 2019. Wu et al. (2019) pointed that people with kin relations not only look alike, they also have similar speaking voices, based on which they proposed to fuse vocal features and facial features for the problem of kinship verification. The method architecture has two sub-networks: vocal network and visual network, as shown in Fig. 10b2. The fusion module applied contrastive learning to learn the fusion scheme. The experimental results demonstrated the effectiveness of multimodal fusion compared to uni-modal (Wu et al., 2019).

Since video-based kinship verification is still a relatively new research topic, only limited research was found in the literature. Although it shows the potential capability of describing more comprehensive features related to kinship when compared to facial images, many issues still remain. We will next provide a summary of video-based kinship verification and discuss the current issues.

### Summary and Discussion

Videos provide an abundance of information that can be leveraged to compensate for the limited temporal information of individual still images. However, varying video quality and multi-source integration bring new challenges to video-based kinship verification research. Both controlled and in the wild videos can enhance the performance of frame-based verification by providing temporal information. The existing benchmarks studied that facial dynamics such as smiles can be another cue for kinship, complementary to facial appearance. Finally, a comprehensive approach is to fuse multiple modalities for kinship verification. However, many problems and unsolved issues still remain in video-based kinship verification.

(1) * Video dataset* Until now, kinship video datasets are very small. Only hundreds of subjects are included in the dataset. This is due to the difficulty of collecting subjects’ facial videos. Unlike facial images that depict single individuals and can be obtained automatically by web crawlers (since facial images sometimes have identity tags), facial videos usually come with multiple non-kin subjects and scene transitions and require careful curation.

(2) * Kin features from videos* Compared with kinship verification from images, research on video analysis is found with limited scale, especially for the unconstrained video-based kinship verification. How to learn kin features from videos (*e.g., *dynamic facial features, multi-modal features such as voice, gait or gestures) is a key research direction.

(3) * 3D facial modal* 3D Morphable Models (3DMMs) (Booth et al., 2018) reconstruct the facial shape from images and videos in unconstrained conditions. 3D face recovery avoids the strict traditional 3D face collection conditions. It reduces the influence of illumination variations brought by the wild environment. Recovering a 3D face in the study of kinship verification remains blank.

## Performance Comparison

In this section, we compare the existing kinship verification methods. Due to a large number of methods tried in FKV, it is not possible to list and compare all of them. Thus, we select representative works and several landmark methods. Tables 4, 5, 6, 7 and 8 summarize the performance of some representative kinship verification algorithms (including feature-based, metric learning-based, and deep learning-based methods) on commonly used benchmark datasets. The widely-used still-image kinship datasets include KinFaceW-I, KinFaceW-II, TSKinFace, and FIW. The depicted video-based kinship datasets are UVA-NEMO Smile, TALKIN, and KIVI.

**Table 4:** Performance comparison (verification accuracy %) of kinship verification methods on KinFaceW-I and KinFace-II datasets

Class			Method	Feature	Metic	KinFaceW-I					KinFaceW-II					Highlights
						FS	FD	MS	MD	Ave.	FS	FD	MS	MD	Ave.	
Traditional method	Feature learning		Periocular features (Patel et al., 2017)	LTP	Cosine similarity	76.3	70.5	73.7	72.5	73.2	79.4	79.0	77.0	72.8	77.1	Studied the contribution of eye region for kinship verification.
			Feature subtraction (Duan and Tan, 2015)	LPQ	Euclidean distance	75.4	63.8	69.9	74.6	70.9	82.4	76.2	76.6	73.2	77.1	A feature subtraction matrix was proposed to remove kinship unrelated parts.
			GInCS (Liu et al., 2016)	Color feature	Euclidean distance	77.3	76.9	75.8	81.4	77.9	85.4	77.0	81.6	81.6	81.4	A illumination robust color space was proposed.
			PML-COV (Moujahid and Dornaika, 2019)	Pyramid feature	L2 vector norm	91.0	84.3	87.1	90.2	88.2	88.6	85.8	87.2	91.0	88.2	Combined the features from multiple resolutions as the pyramid features.
			Feature selection (Cui and Ma, 2017)	HOG, etc.	Mahalanobis distance	**93.6**	84.6	90.4	88.0	89.1	84.0	81.0	84.0	82.0	82.8	To learn the discriminative facial regions by selecting from multiple weak classifiers.
			SP-DTCWT (Goyal and Meenpal, 2020)	DTCWT	Cosine similarity	93.2	**94.4**	**97.1**	**98.0**	**95.9**	**96.2**	**95.4**	**94.6**	**95.0**	**95.3**	Extracts the representative image patches for kinship verification.
	Metric learning	NRML	Basic (Lu et al., 2014c)	LBP, LE, SIFT, etc.	Mahalanobis distance	72.5	66.5	66.2	72.0	69.9	76.9	74.3	77.4	77.6	76.5	To push the neighborhood negative samples away and pull together positive sample.
			PDFL (Yan et al., 2014b)	LBP, LE, SIFT	Euclidean distance	73.5	67.5	66.1	73.1	70.1	77.3	74.7	77.8	78.0	77.0	Project low-level features into hyperspace as discriminative mid-level features.
			S3L (Xu and Shang, 2016a)	LBP, HOG, SIFT	Bilinear similarity	82.4	72.8	**74.6**	79.1	77.2	82.6	73.8	74.1	73.6	76.0	To learn a efficient sparse matric projection for high dimensional kin feature.
			NRCML (Yan et al., 2015)	NRCML	Cosine similarity	61.3	65.5	67.2	62.0	64.9	73.4	70.6	70.8	69.9	73.1	To measure the similarity rather than distance calculation.
			LDCCA (Lei et al., 2017)	HOG	CCA	70.6	65.4	68.6	69.4	68.5	**82.8**	73.6	74.8	76.0	76.8	To capture more mutual information between kinship.
			LM3L (Hu et al., 2017)	LBP, LE, SIFT, etc.	Mahalanobis distance	-	-	-	-	-	82.4	**78.2**	**78.8**	**80.4**	**80.0**	A large margin was proposed to further separate positive and negative pairs.
			ESL (Zhou et al., 2016a)	HOG	Bilinear similarity	83.9	**76.0**	**73.5**	**81.5**	**78.6**	81.2	73.0	75.6	73.0	75.7	A computational efficient method in developing real-world applications.
			SPML (Liu and Zhu, 2017)	HOG	Triangular similarity	**84.3**	75.4	72.4	81.1	78.3	82.4	72.8	75.8	74.0	76.3	Considerd FKV as an asymmetrical problem as differences between kin.
Deep learning			CNN (Zhang et al., 2015)	Architecture	Train	71.8	76.1	84.1	78.0	77.5	81.9	89.4	92.4	89.9	88.4	The first End-to-End deep learning-based
				Task specific	From scratch											kinship verification.
			SMCNN (Li et al., 2016)	Task specific	Pre-trained	75.0	75.0	72.2	68.7	72.7	79.0	75.0	85.0	78.0	79.3	They proposed *L*1-norm as distance loss to train the network in an End-to-End fashion.
			DDML (Lu et al., 2017)	Task specific	From scratch	**86.4**	79.1	81.4	87.0	**83.5**	87.4	83.8	83.2	83.0	84.3	A deep metric learning method maps multiple features into a non-linear plane.
			Appearance+Shape (Zhang et al., 2019)	Resnet	Fine-tuned	81.8	76.6	77.5	77.2	78.3	-	-	-	-	-	Fusing features from both facial appearance modal and shape modal.
			KML (Zhou et al., 2019)	VGG	Pre-trained	83.8	81.0	81.2	85.0	82.8	87.4	83.6	86.2	85.6	85.7	Proposed quadratic similarity metric
				Task specific	From scratch											to analysis the similarity and dissimilarity.
			Attention (Yan and Wang, 2019)	Task specific	From scratch	85.9	81.2	85.2	78.2	82.6	89.8	91.8	93.4	92.8	92.0	Analyze kin clues from specific facial parts rather than whole face.
			AdvKin (Zhang et al., 2020)	VGG-Face	Fine-tuned	76.6	77.3	78.4	86.2	79.6	**91.6**	85.2	90.2	92.4	89.9	Metric learning method combining
				Task specific	From scratch											both contrastive loss and adversarial loss.
			H-RGN (Li et al., 2021a)	ResNet-18	Pre-trained	81.7	78.8	81.4	**88.6**	82.6	90.6	86.8	93.0	**96.0**	91.6	Kin graph taking corresponding feature
				Task specific	From scratch											elements of kinship as the node.
			KIN-MIX (Song and Yan, 2020)	Task specific	From scratch	76.5	75.6	83.5	78.5	78.5	87.2	89.6	90.6	91.2	89.7	They augmented data with linear transform of feature.
			DSMM (Li et al., 2021b)	ResNet-18	Pre-trained	76.7	**81.7**	**89.0**	82.3	82.4	89.8	**92.6**	**95.8**	93.6	**93.0**	Automatically mine discriminative
				Task specific	From scratch											information from negative samples.

(**Task specific** the architecture is built and proposed by the authors to address specific issues. **Pre-trained** the network is trained on the existing datasets. **Fine-tuned** the network weights are initialized with training on existing datasets and then re-trained on the particular datasets. **From scratch** the networks are trained from the beginning on the particular datasets without any external data. Applicable also to Table 5 and Table 6)

Early stages of facial kinship verification research focused mainly on traditional feature extraction methods. The first computational model proposed, tested on the Cornell KinFace dataset, only showed a $65.7\%$ verification accuracy. Subsequently, an increasing number of feature extraction methods were proposed. Several methods based on feature extraction have achieved significant performance improvements. For example, Feature selection method (Cui and Ma, 2017) reached $89.1\%$ and $82.8\%$ average verification accuracy on KinFaceW-I and KinFaceW-II datasets. SP-DTCWT (Goyal and Meenpal, 2020) method achieves the competitive performance and preserves the algorithm efficiency.

Metric learning methods optimize the distance measurement to separate kin and non-kin pairs and improve the verification accuracy. MNRML (Lu et al., 2014a) algorithm is the first remarkable metric learning method that appeared in the kinship verification literature, and it obtained the best performance at that time. On KinFaceW-I and KinFaceW-II, it reached $69.9\%$ and $76.5\%$ accuracy on the average. LM3L (Hu et al., 2014) and SPML (Liu and Zhu, 2017) also show with comparative performance. The performance of metric learning methods is affected by the effectiveness of input features.

**Table 5:** Performance comparison (verification accuracy %) of kinship verification methods on FIW datasets

Method	Architecture	Training	BB	SS	BS	FS	FD	MS	MD	Ave.	Hilights
Benchmark (Robinson et al., 2018)	SphereFace	Fine-tuned	71.9	77.3	70.2	68.5	69.3	69.5	71.8	71.2	Dataset benchmark FIW
ResNet SDMLoss (Wang et al., 2018)	GAN	Pre-trained	72.6	79.4	70.4	68.0	68.3	68.8	71.3	71.2	A pre-trained face de-aging network.
	VGG	Pre-trained									is used to generate a young face.
Dual-VGGFace (Rachmadi et al., 2021)	VGG-Face	Fine-tuned	73.0	65.8	66.9	64.0	65.2	66.2	67.4	66.9	An aux-branch was added to enhance family discrimination.
Unified Approach (Dahan and Keller, 2020)	Task specific	Fine-tuned	**85.9**	**86.3**	**78.0**	**74.9**	**77.4**	**75.6**	**76.9**	**79.3**	Proposed a feature differential loss.

**Table 6:** Performance comparison (verification accuracy %) of kinship verification methods on video datasets

Dataset	Method	Architecture	Training	BB	SS	BS	FS	FD	MS	MD	Ave.	Highlights
KIVI	SMNAE (Kohli et al., 2019b)	Autoencoder		81.3	83.6	82.9	80.0	81.8	77.8	92.3	82.8	Learn the relation between kinship with autoencoders.
TALKIN	Siamese fusion (Wu et al., 2019)	VGG-Face	Fine-tuned	–	–	-	80.0	70.5	73.5	72.5	74.1	Fusing both face and voice modality
		Resnet-50	Fine-tuned									for kinship verification.
UVA-NEMO Smile	Traditional (Dibeklioglu et al., 2013)	–	SVM	63.6	70.0	60.9	60.5	66.1	56.9	57.0	62.2	Combing facial dynamic features with spatio-temporal feature.
	Deep+Shallow (Boutellaa et al., 2017)	VGG-Face	Pre-trained	88.9	94.7	90.1	88.3	93.1	90.5	91.2	91.0	Combing deep feature with spatio-temporal feature.
	Dual AE (Dibeklioglu, 2017)	Task specific	From scratch	**94.2**	**95.7**	**92.6**	**93.4**	**93.8**	**92.2**	**93.6**	**93.7**	Visual transformation of aligned smiling frames.

Deep learning methods proposed later resulted in further performance improvements. For example, DSMM method reached $82.4\%$ and $93.0\%$ average accuracy on KinFaceW-I and KinFaceW-II. With the verification accuracy on small data sets approaching saturation, researchers proposed to move to large-scale kinship verification. Table 5 compares the performance on FIW dataset. Due to different experimental protocol, Table 5 only includes results reported on standard FIW dataset. Further RFIW competition results can be referred to Robinson et al. (2021). Unified approach (Dahan and Keller, 2020) achieved the best performance of $79.3\%$ average accuracy, and there is still a large space for improvement of large-scale kinship verification.

Facial kinship verification is affected by many factors. From the perspective of data acquisition conditions, this includes the recording environment, and the conditions of facial image acquisition, and other factors. From facial attribute point of view, the factors include facial expression, age, and gender. In addition, the size of the kinship dataset is also one of the factors affecting the accuracy. We analyze these factors according to the corresponding experimental results.

(1) *Data collection conditions* Dataset collection can be conducted under two types of conditions: constrained collection in-lab conditions or unconstrained collection in natural environments. Under the constrained environment, taking the UVA-NEMO Smile dataset as an example, the verification accuracy on it is comparatively high since noise brought by environment has been eliminated. The SCCAE method achieves an average verification accuracy of $93.3\%$.

The KinFaceW dataset studies the influences between images from the same photo and different photos. From the verification accuracy listed in Table 4, it can be seen that the KinFaceW-I dataset is relatively lower than that of the KinFaceW-II dataset, where kin images of KinFaceW-II are from the same photo. Similarity of the environment factors provide a clue for kinship verification. The effects of these factors are analyzed in detail in the literature (Bordallo López et al., 2016, 2018).

(2) * Facial expressions* Psychological studies have shown that the performance of kinship verification is influenced by changes in facial expressions (Dal Martello et al., 2015). Kin facial images to be verified have higher recognition rates when they show neutral facial expressions, especially when comparing them to those showing different facial expressions. Results on UVA-NEMO Smile in Table 6 showed that two people with kinship could look similar and have similar smile expressions.

(3) *Age and gender* As people aging, the appearance of their faces varies in structure and texture. These differences affect the inner similarity of kin image pairs, thus reducing the verification performance. The UBKinFace dataset contains facial images of children, young parents, and older parents. From the experimental results in Table 7, we can see that using young parents’ facial images compared to old parents’ facial images shows higher accuracy. In the experiments (Wang et al., 2018), by transforming old images to young parents, the age discrepancy could be mitigated and the average accuracy was improved by about $2\%$.

When considering the gender factor, the experimental results in Table 4 show that same-gender verification, such as FS and MD, turns to have comparatively high performance in contrast to different-gender verification such as FD and MS relationships. Regarding that, Feng and Ma (2021) verified that gender difference could disturb kinship verification performance to some extent. To address this issue, the Gender-FEIT (Feng and Ma, 2021) method was proposed to eliminate the gender gap. Their experimental results demonstrated the method effectiveness by improving the accuracy of FD and MS relations with $1\%$ and $2.8\%$ on KinFaceW-I and $3.4\%$ and $2.9\%$ on KinFaceW-II.

**Table 7:** Verification accuracy ($\%$) on the UBKinFace dataset

Methods	Young parent-child	Old parent-child
DMML (Yan et al., 2014a)	74.5	70.0
MNRML (Lu et al., 2014c)	67.3	66.8
MPDFL (Yan et al., 2014b)	67.5	67.0
KML (Zhou et al., 2019)	75.8	75.2

**Table 8:** Verification accuracy ($\%$) on the second generation relations in the FIW dataset

Methods	GFGS	GFGD	GMGS	GMGD
SphereFace (Robinson et al., 2018)	66.4	66.1	65.4	64.6
ResNet+SDMLoss (Wang et al., 2018)	65.1	65.9	64.9	66.4
TXQDA (Laiadi et al., 2020)	66.8	66.4	65.7	65.2

(4) * Multi-modal data* Recent work (Wu et al., 2016b) has explored fusing both vocal signals and facial images to obtain complementary information, showing a significant performance increase. From their experimental results, vocal modal and visual modal obtained $65.8\%$ and $71.9\%$ average accuracy in a challenging dataset. When fusing both modalities, up to $74.1\%$ average accuracy can be obtained.

(5) * Second generation* When kinship verification takes the facial images of the second generation, the performance drops significantly. Table 8, shows that the performance of the second generation verification is only around $65\%$. As grandparents and grandchildren share less genetic information and have a larger age gap, second-generation kinship verification is more challenging than verification of close relatives.

## Conclusions and Outlooks

FKV is an important yet challenging problem, and has attracted increasing attention. In this paper, we have presented a comprehensive review on FKV from both still images and videos. We have discussed the FKV challenges, existing developments including datasets, evaluation protocols, representative methods, and SOTA performance.

In spite of the recent promising progress achieved by this young field of FKV in the past decade, the technology is still in its early stages and cannot address satisfactorily many of the challenges listed in Sect. 2.2 and many advanced applications. This is evidenced by the poor performance on the large scale dataset FIW collected in the wild, though good performance has been achieved on some small datasets as KinFaceW collected in constrained conditions. However, obviously, the gap between research and real-world application is being narrowed. The current technology is close to bringing the research into some applications with constrained settings. This observation is consistent with those by Robinson et al. (2021). Still, the insufficient data sets pose challenges to efficiently and effectively study facial kinship verification, and deep learning has not reached its full potential yet as face verification.

Finally, we discuss below future research opportunities of FKV, in the hope of providing guidance and insights to interested researchers.

(1) * Large scale dataset establishment for FKV.* The availability of benchmark datasets has played a key role in advancing visual kinship recognition research. Clearly, there is a pressing need to build a large, well-annotated in-the-wild dataset that reflects the true data distribution of facial kinship worldwide and meets the requirement of data-hungry deep learning methods. However, despite the recent progress (*e.g., *the FIW dataset Robinson et al., 2018), such a goal remains unrealized. As we discussed in Sect. 3.3, there are still many problems in the current kinship datasets, such as the following. Firstly, there is the issue of unbalanced ethnicity distribution, which may lead to algorithmic biases like demographic bias (Castelvecchi, 2020). Secondly, current datasets are not large and diverse enough to reflect real-world conditions. Last but not least, most of the current datasets focus on direct descendants without giving full consideration to the height or breadth of the family tree, which is also an important factor for FKV research. Due to privacy, security, and labeling concerns, building a large, diverse, and comprehensive dataset for FKV is much more challenging than for face verification.

(2) * Bias and Fairness* Recently, the AI research community has realized the importance of developing fair and unbiased AI systems (Caton and Haas, 2020; Mehrabi et al., 2021). For instance, facial recognition, Is facial recognition too biased to be let loose (Castelvecchi, 2020), has been shown to have serious demographic bias (Amini et al., 2019; Drozdowski et al., 2020). Kinship recognition relies on people-centric data and also faces such issues. This is especially concerning since kinship recognition systems are intended to be used in critical security applications such as crime scene investigation, border control, or searching for missing children. As we discussed above, datasets play a critical role in FKV. If the training datasets reflect unwanted demographic bias and imbalance, the learned model is unlikely to perform well in the wild. Therefore, taking both algorithm and data biases into consideration in kinship understanding is an important future research direction.

(3) * Accurate features suitable for FKV* Accurate feature representations, suitable for kinship verification, are critical for good FKV performance. However, it still remains a challenging open problem. As we discussed in detail in Sect. 2.2, in contrast to face verification, kin faces are not identical. FKV has large interpersonal variations. The facial similarities of kin faces are often not obvious and vary considerably between different families. All these factors pose great challenges for accurate feature representation. Furthermore, there is another important question: *what features are suitable for kinship verification*? Maybe it is helpful for researchers to be aware of relevant findings in psychology, neuroscience, and anthropology. Finally, facial attributes like gender, age, and skin color may be helpful for FKV. This is the key to learn how to fuse multiple features effectively. Besides, fusing complementary features is also promising in boosting the performance.

(4) * Multimodality* Most current research focuses on kinship verification from facial images, while only a few works consider facial videos. It has been shown that visual kinship verification performance can be enhanced by incorporating multimodal signals such as expressions (Dibeklioglu et al., 2013), voice (Wu et al., 2019), gait (Bekhouche et al., 2020), infrared images (Choe et al., 2017), hyperspectral images (Arya et al., 2015), 3D facial images (Taigman et al., 2014) or facial sketches (Nagpal et al., 2016), among others.

(5) * FKV in the wild* Although many FKV approaches have been proposed, most of them deal with overly easy scenarios. A robust FKV system that can handle the wide intrapersonal variations (such as illumination, expressions, pose, low quality, occlusion, outdoor environments, and natural aging) listed in Sect. 2.2 is still challenging and requires future attention.

(6) * Attack robustness* Currently, deep learning has become dominant in facial image-based recognition systems, including FKV. However, to build an advanced FKV system, increasing the recognition accuracy alone is not sufficient. An FKV system should also be able to resist potential attacks. Recently, it has been shown (Goodfellow et al., 2014; Vakhshiteh et al., 2021) that deep learning-based AI systems are vulnerable to different typical types of attacks, such as adversarial attacks (Madry et al., 2017), and spoofing attacks (de Freitas Pereira et al., 2012). This raises serious concerns in the field of security. However, in FKV, the topic has received little attention (Kumar et al., 2020).

(7) * Privacy awareness* As it is the case for many privacy-sensitive learning tasks such as face recognition and medical image analysis, data privacy in kinship recognition related tasks has also raised some concerns (Kumar et al., 2020). To address them, privacy preserving techniques such as federated learning (Konečnỳ et al., 2016) can be considered. However, to the best of our knowledge, privacy-aware kinship recognition has received very limited attention.

(8) * Kin face synthesis* Kin image synthesis from parents’ faces is helpful in understanding DNA heritage and contributing in solving kinship recognition by enriching existing datasets. Recently, GANs have been shown to generate high fidelity faces (Karras et al., 2017; Kingma and Welling, 2013). Indeed, there are a few works using GANs (Gao et al., 2019; Ghatas and Hemayed, 2020; Sinha et al., 2020) to generate kin faces. However, the existing technology for synthesizing kin faces still remains unsolved. Therefore, we argue the need for future efforts to generate kin faces of varying types, characteristics, and facial expressions.

(9) * Learning with fewer labels* As we discussed above, collecting many facial images for each family is costly. This is especially a concern for problems such as family classification in the wild, with a large number of families. Data-driven techniques like deep learning require a large amount of labeled data for training, which is a current limitation in the field of kinship recognition. Therefore, it is valuable to explore advanced learning methods like domain adaptation, few-shot learning (Wang et al., 2020), and self-supervised learning for kinship recognition.

(10) * Interdisciplinary research* FKV is an important yet challenging problem, with many open issues. It has been studied in several fields, including psychology, anthropology, neuroscience, computer vision, and machine learning. Towards ultimately solving the problem of FKV, we argue that interdisciplinary research should be advocated. For instance, in genetics, automatic computational kinship verification can be applied in exploring facial traits’ computation (Richmond et al., 2018) and genetic problems such as evolutionary patterns of DNA methylation sites (Gokhman et al., 2020).

## Footnotes

1 Siblings are family members of the same generation: Brother-Brother (BB), Sister-Sister (SS), and Sister-Brother (SB). Parent-child relations are first generation: Father-Son (FS), Father-Daughter (FD), Mother-Son (MS), and Mother-Daughter (MD). Grandparent-Grandchild relation belongs to the second generation: Grandfather-Grandson (GFGS), Grandfather-Granddaughter (GFGD), Grandmother-Grandson (GMGS), and Grandmother-Granddaughter (GMGD).

2 A face detection and normalization procedures are typically used to obtain the face in the images.

3 If one expects face verification to achieve age invariance, these pairs of images representing the same individual can also have an age gap and have more significant differences than those of the same age.

4 YouTube is a popular US-based video-sharing website.

5 https://en.wikipedia.org/wiki/General_Data_Protection_Regulation.

6 https://en.wikipedia.org/wiki/Single-nucleotide_polymorphism.

7 https://en.wikipedia.org/wiki/Visa_fraud.
